# Utilization of microbial fuel cells as a dual approach for landfill leachate treatment and power production: a review

**DOI:** 10.1007/s11356-023-30841-w

**Published:** 2023-11-28

**Authors:** Aliyu Ishaq, Mohd Ismid Mohd Said, Shamila Binti Azman, Aliyu Adamu Dandajeh, Gul Sanga Lemar, Zainab Toyin Jagun

**Affiliations:** 1 Department of Water and Environmental Engineering, School of Civil Engineering, Faculty of Engineering, Universiti Teknologi Malaysia, 81300 Johor Bahru, Malaysia; 2 Department of Water Resources and Environmental Engineering, Ahmadu Bello University, Zaria, Kaduna, Nigeria; 3 Department of Biology, Faculty of Science, Kabul University, Jamal Mina, Kabul, Afghanistan; 4 Department of Real Estate, School of Built Environment Engineering and Computing, Leeds Beckett University, City Campus, Leeds, UK; 5 Faculty of Biology, Department of Botany, Kabul University, Kart-e-Char, Kabul, Afghanistan

**Keywords:** Environmental pollutants, Landfill leachate treatment, MFCs, Power generation, Sustainability

## Abstract

Landfill leachate, which is a complicated organic sewage water, presents substantial dangers to human health and the environment if not properly handled. Electrochemical technology has arisen as a promising strategy for effectively mitigating contaminants in landfill leachate. In this comprehensive review, we explore various theoretical and practical aspects of methods for treating landfill leachate. This exploration includes examining their performance, mechanisms, applications, associated challenges, existing issues, and potential strategies for enhancement, particularly in terms of cost-effectiveness. In addition, this critique provides a comparative investigation between these treatment approaches and the utilization of diverse kinds of microbial fuel cells (MFCs) in terms of their effectiveness in treating landfill leachate and generating power. The examination of these technologies also extends to their use in diverse global contexts, providing insights into operational parameters and regional variations. This extensive assessment serves the primary goal of assisting researchers in understanding the optimal methods for treating landfill leachate and comparing them to different types of MFCs. It offers a valuable resource for the large-scale design and implementation of processes that ensure both the safe treatment of landfill leachate and the generation of electricity. The review not only provides an overview of the current state of landfill leachate treatment but also identifies key challenges and sets the stage for future research directions, ultimately contributing to more sustainable and effective solutions in the management of this critical environmental issue.

## Introduction

Landfill leachate is formed when sanitary landfills are utilised to dispose of solid waste from urban areas (Abu-Daabes et al. 2013; Ishaq et al. 2023). Leachate is created by chemical and biological interactions of solid rubbish in landfills, as stated by Abu-Daabes et al. (2013). Leachate from landfills has been shown to include various contaminants, including dissolved organic waste, ammonium, inorganic salt, and other suspended particles (Lu et al. 2009). According to Bhalla et al. (2013), municipal solid wastes dumped on land significantly impact the ecosystem and ecology due to their age, content, and yearly weather fluctuations. Numerous studies have demonstrated that active and decommissioned unlined landfills affect groundwater and surface water by leachate dissipation via soil (Naveen et al. 2018). Landfill leachate contains various contaminants, including toxic metals (Tao et al. 2014) and organic molecules (ammonia nitrogen; Huang et al. 2018). Substrates in microbial fuel cell (MFC) power plants can include everything from organic molecules to living organisms to xenobiotics to heavy metals to inorganic salts and ammonia (Keyikoglu et al. 2021). Activated sludge, oxidation ditches, adsorption processes, trickling filters, lagoon-based treatments, and aerobic-anaerobic digestions are all examples of conventional biological wastewater treatment methods that have run into problems over the past few decades due to issues like high costs, limited space, and high energy requirements (Verma et al. 2021). Using many chemicals and high costs make this approach unfeasible for most situations. While heavy leachate may be deoxygenated via anaerobic treatment, the process has a bad reputation due to its smell. In order to treat leachate and recover energy, anaerobic digestion is one of the most widely employed biological treatment procedures. Instability in the digestion processes and fluctuations in gas output are the main connected issues that might arise from sudden changes in operating parameters (such as organic overloads, over-acidification, ammonia inhibition, etc.). (Wu et al. 2019; Elmaadawy et al. 2020a).

MFC can overcome current technologies’ limitations due to its inexpensive design and construction. For its potential environmental friendliness, MFC is being researched to clean up landfill leachate (Gálvez et al. 2009; Ishaq et al. 2023). MFCs employ microorganisms that clean wastewater while converting organic resources into energy, which might reduce the cost of running an effluent treatment plant (Lu et al. 2009). In recent years, MFCs have emerged as a potential energy-collecting approach. Cost-effective, low-maintenance, powered solely by air, etc. Chemical energy from a wide range of organics (found in landfill leachate) is converted directly into electrical energy by *exoelectrogenic bacteria* in MFCs (Özkaya et al. 2013; Sebastià Puig et al. 2011; Ishaq et al. 2023). MFCs may be utilised to generate power and treat many forms of wastewater (Logan 2010). The MFC framework has made considerable progress in energy recovery and wastewater treatment operations, two of the many shortcomings of traditional treatment systems. The current state of knowledge about MFCs as a treatment tool for leachate and their potential energy-generating applications is the focus of this paper. Since most existing literature deals with wastewater treatment rather than landfill leachate treatment. In light of these problems with traditional treatment methods, innovative treatment methods, such as MFCs, are energetically preferred. Many reviews, as far as we’re aware, have shed light on landfill leachate’s essentials, removal efficiency, and resource recovery (Kelly and He 2014; Pant et al. 2010; Iskander et al. 2016; Mandal et al. 2017; Elmaadawy et al. 2020a). However, modern MFC technology in landfill leachate treatment calls for careful examination.

This article’s goals are to offer a comprehensive analysis of microbial fuel cell technology for nutrient removals in landfill leachate treatment, electricity generation, and water purification. Analyse the synergistic advantages of MFC technology with conventional leachate treatment in terms of boosting treatment and energy recovery, and talk about the prospects for future research and development of MFC technology for efficient treatment (Elmaadawy et al. 2020a).

### Classes of leachate

According to the age of the landfill, there are three types of leachate: young leachate, intermediate leachate, and old leachate (Miao et al. 2014). Landfill leachate can be classified as “young” if it is less than 5 years old, “middle-aged” if it is between five and 10 years old, or “old” if it is more than 10 years old (Jagaba et al. 2021).

Leachate from a young landfill (acid-phase landfills) is often a high-strength effluent with various negative properties. These include a high concentration of volatile fatty acids (VFA) (Neczaj et al. 2005), a high concentration of organic chemicals, a moderate quantity of ammonia (400 mg/L), a low pH, and the presence of various dangerous substances. Leachate from a mature landfill (methanogenic-phase landfills) causes environmental problems despite its low biodegradable organic substance concentration (COD 3000 mg/L), high ammonia concentration (>1000 mg/L), low BOD5/COD ratio (0.1), and high BOD/TKN ratio (Saleem et al. 2018). Landfill leachate aged 30 to 60 years has a COD/TN ratio of 3 to 6 and high biodegradability (Li et al. 2014). Anaerobic decomposition may be to blame for the fall in the proportion of organic pollutants in leachate that can be broken down biologically as landfills age. According to studies (Aziz et al. 2011), refractory organics are less abundant in fresh leachate than in older leachate.

#### Leachate degradations

Leachate is an aqueous effluent that results from the breakdown of organic components in waste by bacteria and physicochemical processes. It is an extremely contaminated wastewater that varies depending on the quantity of garbage it contains and how much of it has degraded and broken down. Seasonal variation, waste properties, moisture content, acidity, and temperature all have an impact on leachate deterioration and stabilization (Renou et al. 2008; Schiopu and Gavrilescu 2010). The decomposition of garbage is affected by a plethora of chemical and biological reactions that take place in landfills (Jagun et al. 2022). Leachate’s physical properties are affected by the concentration of inorganic particles like iron and lead and their temperature and colour (Slack et al. 2005). Landfills degrade garbage via at least five different phases, each of which is followed by the emission of a specific combination of chemicals and gases. Aerobic: Water and carbon dioxide are the two most abundant byproducts, with the latter either being released as a gas or dissolved in water to form carbonic acid, the acidic component of leachate. Carbon dioxide, hydrogen, ammonia, and organic acids are all examples of acidogenic chemicals, whereas acetic acid and its derivatives and carbon dioxide and hydrogen are all examples of acetogenic substances. Methanogenic: the typical composition of landfill gas is 60–40% methane and carbon dioxide. Carbon dioxide and water are the key ingredients in an aerobic environment (Flimban et al. 2019).

**Fig. 1:**
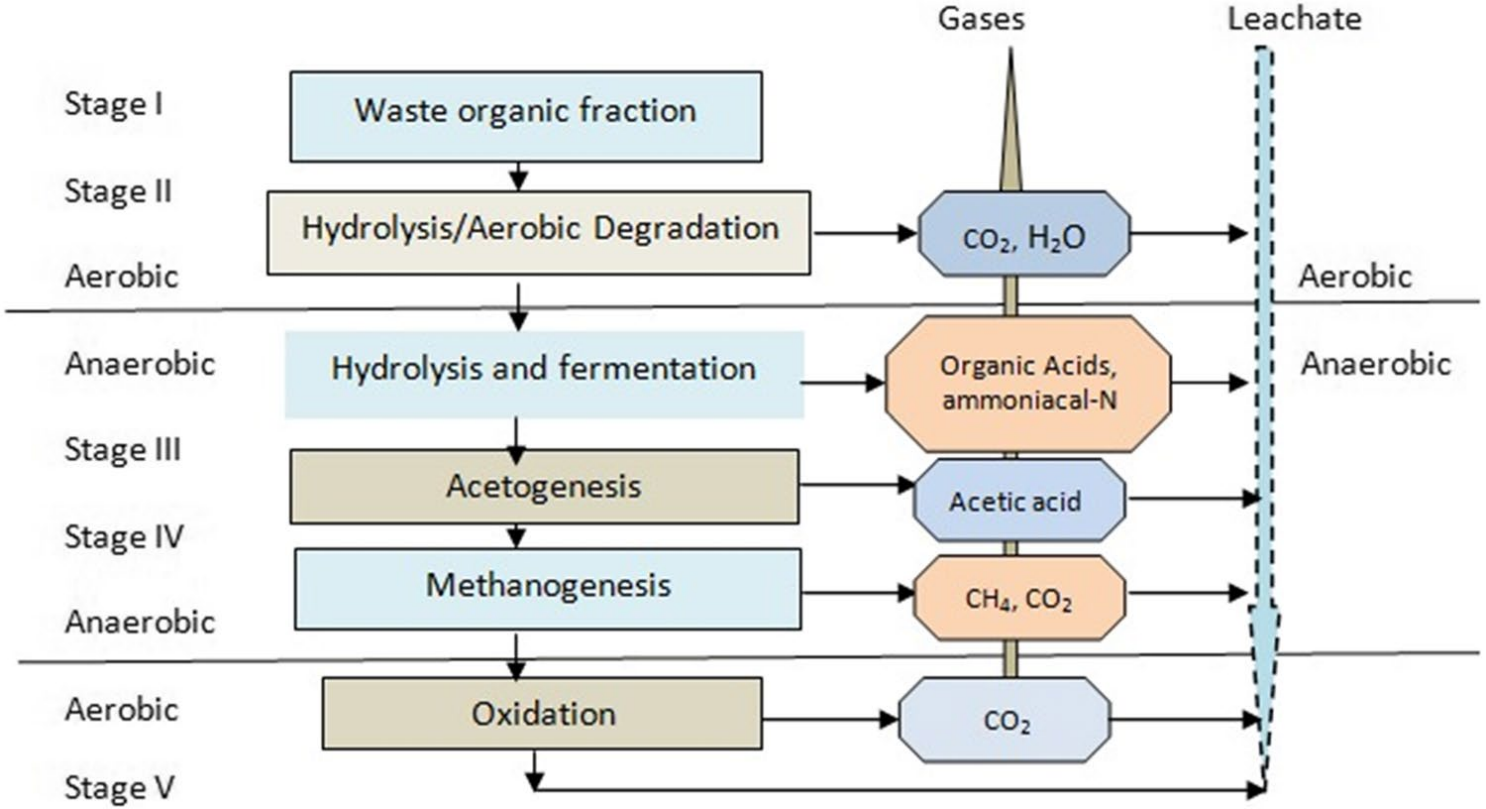
Schematic diagram of leachate biological Degradation processes (Adhikari 2015)

Various landfill regions may be in differing states of decomposition at any given time. These processes may continue even after the landfill has been covered or closed (Christensen et al. 2001; Kjeldsen et al. 2002). There are four distinct phases in the decomposition of organic waste, as shown in Fig. 1, each with unique properties and factors (Schiopu and Gavrilescu 2010). The resultant leachate can be categorized based on these factors. (1) Chemical oxygen demand (measured as COD), which includes:

i) Ammonia nitrogen ($${\textrm{NH}}_4^{+}$$–N).
ii) Dissolved solids.
iii) Suspended solid.
iv) Xenobiotic organic compounds (XOCs).
v) Heavy metals.
vi) Salts.

Different parts of a landfill are at various stages of decay at any given moment. These processes may persist even after the landfill has been covered or closed (Christensen et al. 2001; Kjeldsen et al. 2002). According to Schiopu and Gavrilescu (2010), the breakdown of organic waste may be broken down into four separate stages, each with its own characteristics and contributing variables. Based on these characteristics, the resulting leachate may be sorted. Which is comprised of the following:

- Leachate from landfills typically contains larger amounts of inorganic macro-compounds than surface water does, while the concentration of specific macro-components can vary with landfill age. Some of the most common inorganic compounds include: ${NH}_4^{+}$, $nitric\ and\ nitrate\ oxides\ \left({NO}_3^{-}\right)$, $nitrate\ ions\ \left({NO}_2^{-}\right)$, ${SO}_4^{2-}$, Cl-, ${PO}_4^{3-}$, sodium (*Na*^+^), potassium (*K*^+^), calcium (*Ca*^2+^), magnesium (*Mg*^2+^), iron (*Fe*^2+^) and hydrogen carbonate (HCO3-) (Robinson 2007). Some cations (including calcium, magnesium, and iron) are absorbed by organic molecules and precipitated out with anions due to the high pH in methanogenic leachate. This results in a leachate with a decreased cation content. Asadi (2008) notes, leaching washes away several macro-components, including Cl-, *Na*^+^, and *K*^+^, leaving them with little time to undergo sorption, precipitation, or complexation into other compounds. Due to the lack of alternative ammonia degradation processes, leaching has been proposed as a potential mechanism for ammonia reduction in the methanogenic phase. Therefore, ammonia has been found to be the major component of leachate over the long term, according to various studies. The range of 50–200 mg/L of ammonia has positive effects on anaerobic processes at higher pH levels, whereas concentrations of 1500–3000 mg/L are inhibitory, according to research by Kale et al. (2010). Microorganisms are killed off at ammonia concentrations exceeding 3000 mg/L (Kale et al. 2010). In addition, leachate with a nitrate origin (such as sewage, fertilizer, farm, animal waste, food waste, etc.) is readily observable. Microbial conversion of sulfate to sulfide occurs during the methanogenic phase (Bhalla et al. 2012), leading to reduced sulfate concentrations.
- Contaminants of emerging concern (CEC): The category of rising concern includes primarily unregulated substances, including micropollutants, pharmaceuticals, and personal care products (PPCPs), as well as endocrine-disrupting compounds (EDCs). These substances have been observed in the natural environment (Qi et al. 2018). Propp et al. (2021) have documented many forms of cation exchange capacity (CEC) in leachate derived from a historical landfill. These include polyfluroalkyl substances (PFAS), perfluorooctanoic acid (PFOA), organophosphate esters (OPE), substituted phenols, bisphenols, perchlorate, and pharmaceutical compounds. The study conducted by Propp et al. (2021) reported the highest observed value of 12.7 μg/L for per- and polyfluoroalkyl substances (PFAS). Additionally, the investigation found considerably elevated concentrations of other substances, including OPE, sulfamic acid, cotinine, and bisphenols. In a similar vein, a total of fifty-eight CEC (Contaminants of Emerging Concern) compounds were identified in samples of landfill leachate collected from Greece. Notably, the substances Bisphenol A, valsartan, and 2− OH-benzothiazole exhibited the highest average concentrations among the observed CEC compounds. The study conducted by Nika et al. (2020) found that pharmaceuticals, industrial chemicals, and agrochemicals were the primary types of contaminants of emerging concern (CEC) that were observed. Qi et al. (2018) have shown that the kinds of CECs most commonly studied in Chinese landfill leachate include phthalate esters and PPCPs. Nine different contaminants of emerging concern (CEC) have been seen to exhibit a wide range of concentrations, spanning from 0.03 to 4500 μg/L.
- Xenobiotic organic compounds (XOCs): Xenobiotic organic chemicals (XOCs) are often found in low amounts, typically below 1 mg/L for specific substances. Benzene, toluene, phenols, chlorinated aliphatics, phthalates, and halogenated hydrocarbons such as tetrachloroethylene and trichloroethylene are among the XOCs that have been identified (Propp et al. 2021). The main sources of these pollutants consist of home and industrial chemicals, as well as pesticides and fertilizers. The level of contaminants in leachate is influenced by waste mix, landfill technique, and age. The extensive investigation of monoaromatic hydrocarbons, including benzene, toluene, xylenes, and halogenated hydrocarbons, has been conducted due to their detrimental effects on both the environment and human health (Adhikari 2015). The analysis of monoaromatic hydrocarbons is straightforward.
- Heavy metals, salts: leachate commonly contains a range of heavy metals, including zinc (Zn), nickel (Ni), lead (Pb), copper (Cu), chromium (Cr), and cadmium (Cd). Additionally, there are metalloids such as arsenic (As), selenium (Se), mercury (Hg), and cobalt (Co), but these are typically found in minimal quantities. According to Ishak et al. (2016), the extended persistence of heavy metals in leachate can be attributed to their non-biodegradable and soluble characteristics. Consequently, these substances can undergo bio-magnification as they accumulate progressively along the food chains, resulting in many detrimental consequences for both humans and other organisms (Wijesekara et al. 2014). The primary sources of heavy metal discharge into leachate are batteries, automobile components, electrical wire, alloys, paints, lamp filaments, and ceramics (Trabelsi et al. 2013). The concentration of heavy metals in landfill leachate is typically minimal. However, there may be modest variations in concentration according to the degradation phase of the landfill.
- Dissolved organic matter (DOM): DOM constitutes a significant constituent within landfill leachate, and it exhibits interactions with various inorganic and organic contaminants, including heavy metals, as demonstrated by Huo et al. (2008). DOM refers to the proportion of organic material that is capable of traversing a filtration membrane with a pore size of 0.45 μm, including a diverse array of molecular weights and sizes. A study conducted by He et al. (2006) identified many major DOM components that are found in landfill leachate at significantly elevated levels. These components include amino acids, volatile fatty acids (VFA), hydrophilic acids, as well as fulvic-like and humic-like chemicals. The presence of many functional groups in DOM, such as carboxylic, phenolic, alcoholic, hydroxyquinone, and carbonyl groups, contributes to its high functional capacity in the environment. These functional groups enable DOM to interact with other compounds present in leachate (Kjeldsen et al. 2002; Vithanage et al. 2017). According to Vithanage et al. (2017), a greater presence of humic-like compounds indicates that the leachate is in an intermediate state of degradation.
- Conversely, a significant concentration of volatile fatty acids suggests an earlier acetogenic phase. DOM has the potential to impact the transit and evolution of heavy metals such as cadmium (Cd), copper (Cu), mercury (Hg), lead (Pb), manganese (Mn), nickel (Ni), and zinc (Zn) through the formation of complex species (Robinson 2007). Significantly, the phenomenon of the dark brown colour of leachate can be attributed to the presence of humic and fulvic chemicals, which can form complexes with ferric hydroxide colloids (Wijesekara et al. 2014). Hence, the determination of DOM is commonly conducted by the measurement of several bulk properties, including biological, biochemical oxygen demand (BOD), chemical oxygen demand (COD), total organic carbon (TOC), volatile fatty acids (VFA), and specific chemicals like as methane (Kjeldsen et al. 2002). In general, elevated levels of biochemical oxygen demand (BOD) and chemical oxygen demand (COD) in leachate are indicative of a substantial presence of DOM. Conversely, a low BOD/COD ratio suggests lower concentrations of volatile fatty acids and larger quantities of humic and fulvic-like compounds (Adhikari 2015).

**Fig. 2:**
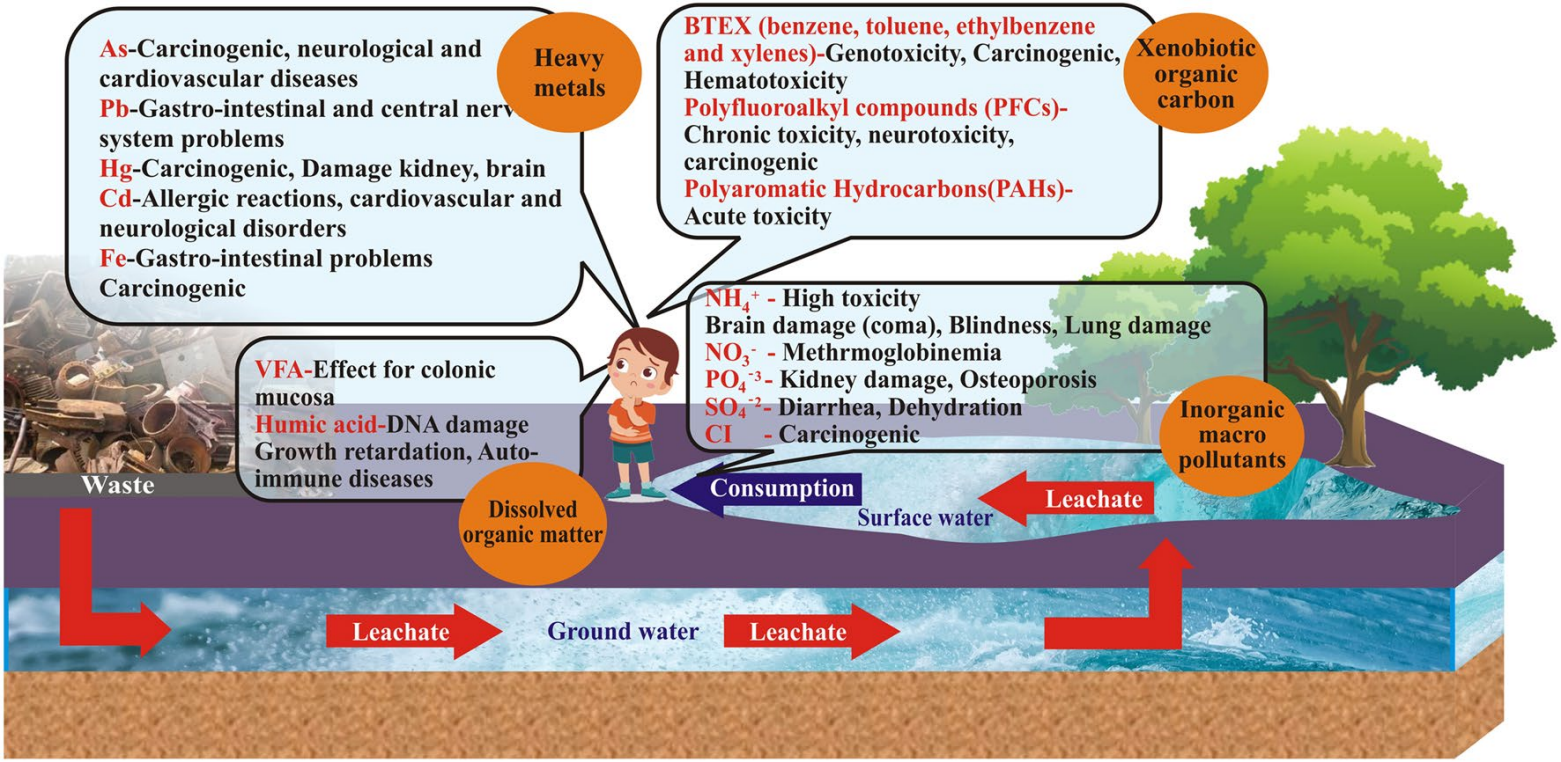
Impacts of landfill leachate components. (Wijekoon et al. 2022)

In instances when landfill leachate is not appropriately managed via collection, treatment, and safe release, it can give rise to many environmental concerns, including soil contamination, surface water pollution, groundwater contamination, and potential risks to human health (Jagun et al. 2022). The potential adverse impacts of landfill leachate on ecosystems and human health, as illustrated in Fig. 2, can be attributed to the elevated concentrations of ammonia, heavy metals, and certain organic compounds (Volatile Organic Compounds (VOCs)). Therefore, leachate is seen as possessing the capacity to induce eco-toxic effects that exert stress on many components of the ecosystem. The presence of significant quantities of xenobiotic organic compounds (XOCs) and heavy metals in leachate has the potential to result in the accumulation and bio-magnification of these substances in the tissues of animals across different trophic levels within food chains. This accumulation can lead to the development of carcinogenic effects, as well as acute and genotoxicity outcomes (Mukherjee et al. 2015; Toufexi et al. 2013). The illustrations of the pollutants are clearly shown in Fig. 2.

### Composition of landfills

The composition of leachate is affected by many factors, such as the waste’s type and quantity, the grinding level, the compaction level, the degradation processes (hydrolysis, adsorption, biodegradation, speciation, dissolution, dilution, ion exchange, redox, contact time, partitioning, precipitation gas, heat generation, and transport), the humidity of the waste, the climate, the hydrology of the storage site, the temperature of the waste while stored, and the le (Jagaba et al. 2021). Other features of the system include liquid waste co-disposal, water recirculation for irrigation and trash processing (Jagaba et al. 2021b). Leachate from landfills is influenced by a number of factors, including landfill age, solid waste components, rainfall rate, and landfilling technique (Remmas et al. 2018). Factors such as the waste’s substance, age, and size impact the quantity of leachate generated, while the landfill’s geology and weather conditions determine the degree to which rubbish is compacted (Choudhury et al. 2021). Alcohols, humic and fulvic acids, and volatile fatty acids are all examples of organic compounds, inorganic compounds (e.g., Ca^2+^, Na^2+^, K^2+^, Mg^2+^, Fe^2+^, Mn^2+^, ${\textrm{NH}}_4^{+}$-N, ${\textrm{SO}}_4^{2-}$, Cl and HCO_3_ ), heavy metals (e.g., Cd, Pb, Cr, Ni, Hg, Cu, and Zn), and persistent organic compounds, pathogens, medicines, cyanides, total dissolved salts, NH_3_-N, total alkalinity, COD, total hardness, solvent, and carcinogens with an unpleasant odour are all present in leachate (Aziz et al. 2011; Neczaj et al. 2005; Yong et al. 2018; Jagaba et al. 2021).

### Characteristics of landfill leachate

The properties and composition of leachate can be affected by several factors, such as the waste’s type and quantity, the degree to which it was crushed and compressed, the decomposition process (hydrolysis, adsorption, decay, speciation, refreezing, dilution, ion exchange, redox, contact time, separating, precipitation gas, heat dissipation, and transport), and the decomposition time (Aluko and Sridhar 2014; Mojiri et al. 2016). This is due to the fact that leachate varies greatly in terms of its composition and features as a result of the dynamic and complex nature of these elements. Therefore, it is difficult to manage and treat leachate without taking into account these aspects in order to lessen the negative effects on the environment and guarantee efficient treatment. When cleaning up leachate, it’s common to use a combination of physical, chemical, and biological methods, depending on the nature of the pollutants in question. Leachate undergoes fast alterations after its production, the nature of which is determined by its source and how old it is. Leachate strength changes as landfill disposal time progresses after closure. After only 2 days in the waste holding cell, leachate from incinerators and transfer stations can still preserve its quality (Remmas et al. 2018; Jagaba et al. 2021). The final composition of landfill leachate is made up of dissolved organic molecules (alcohols, humic, fulvic, and VFA), inorganic compounds (e.g., Ca^2+^, Na^+^, K^+^, Mg^2+^, Fe^2+^, Mn^2+^, ${\textrm{NH}}_4^{+}$-N, SO_4_^2−^, Cl^−^, and HCO_3_), and heavy metals (e.g., Cd, Pb, Cr, Ni, Hg). Due to specific waste qualities, leachate may produce *NH*_3_ − *N*, total alkalinity, COD, total hardness, solvent, malignancy, and a terrible odour. The environment, climate, landfill operation, landfilling technology, waste age, and weather conditions significantly influence each quality (Remmas et al. 2018). The leachate is classified as young (less than 5 years old), intermediate (5 to 10 years old), or old (more than 10 years old), depending on how long it has been lying in a landfill (Jagaba et al. 2021). Leachate often contains significant quantities of ammoniacal nitrogen (*NH*_3_ − *N*), chloride, and sulphate, as well as trace levels of heavy metals and other organic components (Lebron et al. 2021). Young landfill leachate (acid-phase landfills) has a lower BOD5/COD ratio and a lower concentration of biodegradable organic components (COD 3000 mg/L) than older landfills (Ying et al. 2012). In mature landfill leachate (methanogenic phase landfill), which has a high proportion of molecular weight organics, there is a low percentage of biodegradable organic compounds, rubbish, and so on (Ying et al. 2012). The middle-aged landfill, on the other hand, has both a very low concentration of ammonia (0.10) and a very high concentration of ammonia (>1000 mg/L), both of which are global concerns due to their fertilising and destructive impacts. The fraction of biodegradable organic pollutants in leachate decreases with landfill age, which may be connected to anaerobic decomposition (Ying et al. 2012). Organic compounds, both biodegradable and nonbiodegradable, and heavy metals, phenols, *NH*_3_ − *N*, sulphide, and phosphate are abundant. The landfill leachate characteristics with emerging contaminants are described in Table 1.

**Table 1:** Landfill leachate physicochemical composition with emerging contaminants

Landfill leachate characteristics						
Acetogenic leachate			Methanogenic leachate			
S/N	Parameters (mgL^−^1)	Young age (years) < 5	Middle age (years) 5–10	Mature age (years) (> 10)	FEPA standard 1991	References
1	pH	< 6.5	6.5–7.5	> 7.5	6–9	Renou et al. (2008)
2	COD	> 10,000	4000–10,000	< 4000	-	Renou et al. (2008)
3	BOD	0.5–1.0	0.1–0.5	< 0.1	30	Renou et al. (2008)
4	NH_3_-N	< 0.4	NA	> 4000	-	Renou et al. (2008)
5	TOC/COD	< 0.3	0.3–0.5	> 0.5	-	Statom et al. (2004)
6	Heavy metals	Low-medium	Low	Low	< 1	Kamaruddin et al. (2017)
7	Total Kjeldahl nitrogen	1500–4500	400–800	75–300	-	Renou et al. (2008)
8	P	100–300	10–100	-	-	Mavakala et al. (2016)
9	Biodegradability	High	Medium	Low	Low	Aboyeji and Eigbokhan 2016
10	Alkalinity	8000–18,000	4500–6000	-	-	Scott et al. (2005)
11	Conductivity (μs.cm)	15,000–41,500	6000–14,000	-	-	Wijesekara et al. (2014)
12	$two{\textrm{so}}_4^{2+}$	500–2000	200–1000	50–200	-	Wang et al. (2002)
13	Ca^2+^	10–250	6200	5500	200	Xaypanya et al. (2018)
14	Mg^2+^	40–1150	-	-	200	Xaypanya et al. (2018)
15	Fe^2+^	500–1500	500–1000	100–500	10	Xaypanya et al. (2018)
16	Zn^+^	100–200	50–100	10–50	<1	Xaypanya et al. (2018)
17	Cl^-^	1000–3000	500–2000	100–500	-	Bove et al. (2015)
18	Total dissolved solids (TDS)	10,000–25,000	5000–10,000	2000–5000	2000	Renou et al. (2008)
19	VFA	VFA (80%)	VFA (5–30%)	HA and FA (80%)	-	Tejera et al. (2019)
20	VOCs	0–3	0–2.5	0–2.5	-	Chiemchaisri et al. (2019)
21	Emerging contaminants ▪ Indole ▪ Isoquinoline ▪ Menthol ▪ 3-Beta-coprostanol ▪ Bromacil ▪ Caffeine ▪ Cholesterol ▪ Diethoxynonyl phenol	- - - 0.00500–0.600 - 0.0140–0.0800 0.00500–1.50 -	0.08–15.7 0.220–9.90 0.819–3.52 2.00 1.00–11.2 0.100–0.300 2.00 1.48–10.0	- - - - - - - -	- - - - - - -	Andrews et al. (2012)
22	Total coliform		-	-	400	Bhalla et al. (2013)

*Unit in mg.L^−1^ not applicable to pH parameter. *P*, phosphorus; SO_4_-Sulphate

( - ) Not measured

### The negative impact of leachate on groundwater contamination

Groundwater contamination from landfill leachates is a lengthy and progressive process. Leachates from landfills account for 10% of all municipal waste (Aluko and Sridhar 2014), and about 70% of all rubbish is biodegradable. While the leachate treatment plant may handle some leachate, some may leak out of the landfill and into the surrounding soil and groundwater. If not properly managed, leachate can contaminate the surface and groundwater (Aluko and Sridhar 2014). Due to its components' harmful and persistent nature, heavy metals and ammonia produce a continual shift in input and toxicity (Trabelsi et al. 2013). The transport mechanisms and persistence of leachate compounds in groundwater create long-term contamination issues, making remediation costly and challenging. Groundwater contamination not only endangers human health but also jeopardizes aquatic ecosystems and drinking water sources (Hussein et al. 2021). The release of leachate into soil and aquifers seriously threatens human health and the environment (Remmas et al. 2018). When dumped in a natural setting, raw leachate can seep into the earth and surrounding water sources, severely contaminating both (Longe and Balogun 2010). This negatively affects the soil and the entire biological system, including humans(Yong et al. 2018). The rate of leachate leakage differs significantly between the landfilling phase, the covering phase, and the entirely covered phase because the leachate depth varies with penetrating rainfall. Most developed countries, therefore, have legislation for treating hazardous elements of leachate before disposal (Ishaq et al. 2022) to prevent contamination of water resources and major and chronic toxicity intrusion. Ammonia, metals, colour, dissolved solids, organic chemicals, and inorganic compounds are the most common contaminants in landfill leachate. Due to its high concentration in landfill leachate, ammonia has become a critical issue among many contaminants, posing significant environmental risks and negatively impacting human health. The leachate plume impacts hydrogeological processes in the aquifer, extending hundreds of meters when mixed with the unconfined aquifer (Mor et al. 2016). Therefore, it’s important to study concentrations and find the best treatment method. This review article relied on a narrative literature search to identify the current findings on the topic.

### Standard regulations for leachate management and discharge

Landfill can become an underlying source of pollution due to the danger of leachate penetration into the soil and groundwater if it is not disposed of effectively. As a result, the created leachate must be gathered and handled carefully before being released into the natural habitat. According to Tsilogeorgis et al. (2008), proper management of a landfill site may significantly cut down on the amount of leachate created as well as the volume of it. However, leachate cannot be removed entirely. According to Aftab et al. (2020); Deng et al. (2020), if untreated raw leachate is disposed of, it can become a major source of water pollution. Because leachate may create major environmental problems, it must be collected and appropriately treated before being released into the environment (Deng et al. 2020). Environmental and economic considerations (Jagaba et al. 2021) and the technology applied to remove leachate may explain the variation in standard limit values across locations. To meet discharge standards, leachate treatment becomes a significant challenge (Trabelsi et al. 2013). The regulations governing the management and discharge of leachate vary across different countries and regions, being typically under the jurisdiction of local environmental agencies and authorities. Nevertheless, there exist certain shared principles and standards that often serve as guidelines for the management and discharge of leachate from landfill sites. Several general aspects and standards pertaining to leachate management include the following:

1. Effluent standards: Regulations frequently stipulate the maximum permissible concentrations of various contaminants in leachate effluent. These pollutants include factors like Chemical Oxygen Demand (COD), Biological Oxygen Demand (BOD), Total Suspended Solids (TSS), pH levels, heavy metals, and specific organic and inorganic compounds. These standards are established to safeguard the quality of surface water and groundwater.
2. Discharge permits: Landfill operators typically must obtain permits for the discharge of leachate. These licenses outline specific circumstances, monitoring prerequisites, and release limits that must be followed to comply with environmental regulations.
3. Monitoring and reporting: Regular monitoring of the quality and quantity of leachate is often mandated. Landfill operators are obligated to submit reports to regulatory authorities that provide detailed accounts of the results obtained from monitoring efforts. This information aids in ensuring compliance with discharge standards.
4. Treatment requirements: Depending on the characteristics of the leachate and its potential impact on the environment, treatment may be necessary prior to discharge. Treatment techniques may involve physical-chemical treatment, biological treatment, or a combination of these approaches.
5. Landfill design and liner systems: Regulations frequently establish design requirements for landfill liners and leachate collection systems with the aim of minimizing the migration of contaminants into groundwater. Proper construction and maintenance of liners are of paramount importance in preventing leachate leakage.
6. Buffer zones and setbacks: Regulations may necessitate the implementation of buffer zones or setbacks between landfills and vulnerable receptors, such as drinking water wells, rivers, or residential areas, in order to safeguard against contamination.
7. Closure and post-closure care: Regulations may also govern the closure and post-closure care of landfills, including the management of leachate during and after the operational phase of a landfill.
8. Environmental impact assessments: In the case of new landfill projects or expansions, it may be required to conduct environmental impact assessments to evaluate potential impacts on the surrounding environment and to devise appropriate mitigation measures.
9. Public notification: Certain regulations may mandate public notification and consultation regarding landfill operations and leachate management, particularly when there is a potential for environmental or health impacts.
10. Emergency response plans: Landfill operators may be obligated to develop and maintain emergency response plans in the event of accidents or unexpected releases of leachate.

### Several methods of landfill leachate treatment worldwide

**Fig. 3:**
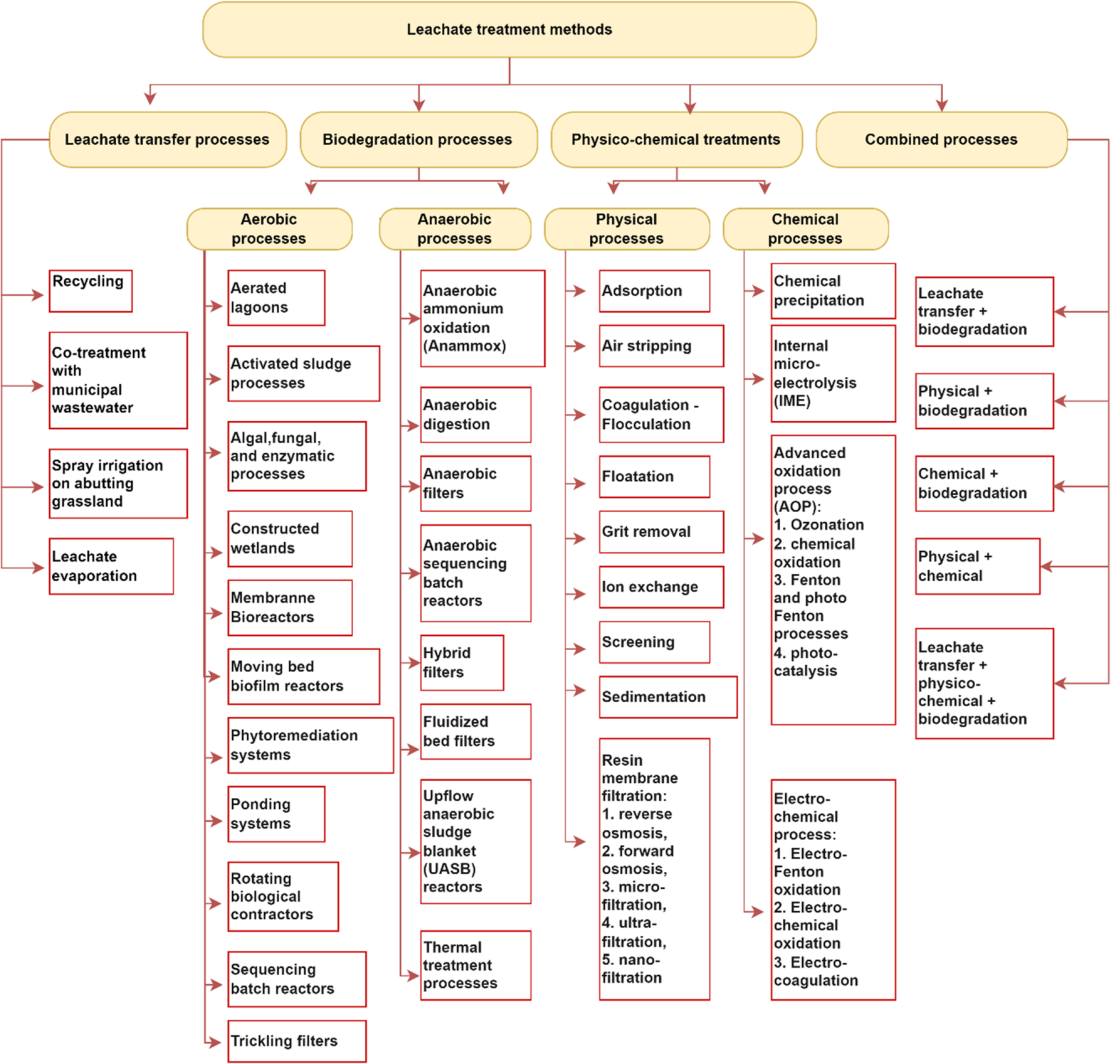
Different methods of Leachate recovery, reuse and degradation. Source: (Jagaba et al. 2021)

Several methods exist for treating landfill leachate, each aiming to be effective and compliant with relevant regulations. Physical-chemical (flotation, coagulation/flocculation, adsorption, chemical precipitation, air stripping, pH adjustment, chemical oxidation, ion exchange, and electrochemical treatment) and biological (activated sludge, aerobic and anaerobic stabilisation lagoons, and biological filters) methods are all used. There are advantages and disadvantages to these treatments and their effectiveness. The success of leachate remediation is increased when multiple approaches are combined, as proven in several studies (Biki et al. 2021; Jagaba et al. 2021). Combining leachate treatment methods has increased the amount of COD, NH_3_, organic matter, and other pollutants that may be removed. The leachate recovered and collected from a landfill must be managed appropriately, utilizing one or more of the three applicable procedures listed: Treatment on-site, dumping into sewage systems, and transport away from the site for treatment offsite are all choices. These three treatment approaches can be subdivided into sub-methods, as shown in Fig. 3. These methods are discussed extensively in the subsection.

#### Leachate transfer processes

Recycling: Partial treatment and disposal of leachate-by-leachate recycling looks to be one of the cheapest options at well-designed and -operated landfill sites. During recycling, pollutants and impurities are often removed through a series of treatment procedures, rendering the leachate appropriate for uses like irrigation, industrial activities, and even as a water supply. The two main components of this recycling strategy are collection and pre-treatment. Recycling leachate is an effective strategy for dealing with landfill leachate, reducing adverse environmental effects, and protecting scarce water supplies. To promote a more sustainable and circular waste management system, leachate may be converted into a resource using suitable treatment technologies (Tatsi et al. 2016). Leachate recirculation has been shown to increase the moisture contents within a reactor system, providing sufficient distribution of nutrient elements and enzymes among methanogens and solids/liquids, leading to a notable decrease in methane yield and COD (Carvajal-Flórez and Cardona-Gallo 2019; Abdel-Shafy et al. 2023). Landfill leachate: Sources, nature, organic composition, and treatment: An environmental overview. Ain Shams Engineering Journal, 102293.). After recirculation, Ghosh et al. (2017) found that the COD in an anaerobic pilot plant dropped by 63 to 70%. The stabilisation time required was also shown to be reduced to two to three years via recirculation. However, significant recirculation rates may have a harmful effect on anaerobic degradation. It was also determined that methanogenesis inhibition due to high levels of organic acids (pH less than 5), which poison the methanogens, may result from leachate recirculation (Ghosh et al. 2017). Acidic conditions, saturation, and ponding can all result from excessive leachate recirculation.

Co-treatment with municipal wastewater: Low biodegradability and heavy metals of the organic inhibitory compounds in the leachate have long cast doubt on the usefulness of such a process, suggesting instead that treatment efficiency would suffer and effluent concentrations would rise (Carvajal-Flórez and Cardona-Gallo 2019; Abdel-Shafy et al. 2023). The presence of nitrogen in leachate and phosphorus in sewage necessitated urgent volumetric optimisation of leachate’s part within the overall wastewater. Filling, anaerobic, aerobic, and settling comprise a sequencing batch reactor (SBR), which is proposed as a combined treatment approach (Contrera et al. 2014). With a sewage-to-leachate ratio of 9:1, roughly 95% of BOD and 50% of nitrogen on a cycle basis were removed. An increase in the proportion of landfill leachate to municipal wastewater was determined to decrease COD and BOD concentrations. Meanwhile, adding powdered activated carbon (PAC) can considerably improve effluent quality (Deng et al. 2018).

Leachate evaporation: The liquid produced by landfills may be treated by exposing it to solar radiation in evaporation ponds or basins under strict supervision. Solar light warms the leachate as it spreads across a broad region, and this, in turn, causes water to evaporate into the air. Although it can release harmful gases and odours into the atmosphere, in addition to weather-dependence affects its effectiveness, and disposing of the residue is problematic. Pollutants and dissolved solids in the leftover leachate are concentrated by this procedure, making it more manageable for further treatment or disposal. As a result of faster evaporation rates in areas with high temperatures and low humidity, the amount of leachate may be reduced, and the environmental implications of uncontrolled leachate discharge can be mitigated. However, to comply with local rules and environmental requirements for responsible waste management, it is crucial to consider the possibility of extra treatment of the concentrated leachate residue.

Spray irrigation on abutting grassland: The land application technique of spray irrigation, which involves the dispersal of landfill leachate into adjacent grassland, is employed with the objective of effectively managing leachate and facilitating its controlled dispersion. This methodology has the potential to yield advantages such as reducing leachate levels and mitigating the risk of groundwater pollution while concurrently offering an effective method for the disposal of this liquid waste. The leachate is applied onto the grassland, facilitating its absorption and subsequent natural treatment by the soil and plants. This process has the potential to assist in the elimination of certain toxins. Nevertheless, it is important to implement efficient management and monitoring practices in order to prevent the process from surpassing the soil's ability to absorb leachate, which might lead to run-off or excessive pollution. Furthermore, it is important to thoroughly evaluate the potential ecological and agronomic consequences on the grassland and its surrounding ecosystem. This underscores the need to achieve a harmonious equilibrium between waste disposal practices and environmental preservation.

#### Biodegradation process (biological treatment of leachate)

According to Oller et al. (2011), biological treatment processes have proven to be really effective in getting rid of high concentrations of Chemical Oxygen Demand (COD) and Biochemical Oxygen Demand (BOD) from leachate. The two types of treatment processes, aerobic and anaerobic, are classified based on the requirement of oxygen. In the case of aerobic treatment systems, the presence of oxygen plays a crucial role in breaking down the pollutants, resulting in the production of carbon dioxide, solid biological byproducts, and sludge, as explained by Grady et al. in 2011. On the other hand, in an anaerobic process, the organic matter goes through a conversion process that results in the production of biogas, which is mainly made up of carbon dioxide, methane, and biological sludge. The effectiveness of biological processes in removing organic and nitrogenous materials from immature leachate, characterized by its youthfulness, has been well-documented, particularly when the ratio of BOD to COD is relatively high, exceeding 0.5, as observed by Naveen et al. (2018). Nonetheless, it is crucial to acknowledge that the presence of refractory compounds such as humic and fulvic acids can potentially limit the overall effectiveness of the biological treatment process over time, as highlighted by Abbas et al. (2009). Biodegradation occurs when microorganisms break down organic substances into carbon dioxide, sludge, and biogas (a mixture mainly composed of CO_2_ and CH_4_) in an aerobic environment (Abdel-Shafy et al. 2014). The removal of leachate laden with high levels of BOD is a widespread application of a biological treatment, primarily distinguished by its excellent cost-effectiveness, dependability, and simplicity. Biological activities successfully treat high BOD/COD ratio values (higher than 0.5) because they help remove nitrogenous and organic materials from young leachate (Tatinclaux et al. 2018). Humic and fulvic acids are examples of refractory substances that may reduce the treatment’s efficacy over time.

Suspended-growth biomass: The term “suspended-growth biomass” describes a population of microorganisms, primarily *bacteria and protozoa*, that are actively developing and freely floating in a liquid media as part of a wastewater treatment process. These *bacteria* are essential to biodegradation because they feed on and decompose the organic materials, nutrients, and pollutants that are found in wastewater. The suspended-growth systems used in wastewater treatment facilities, such as activated sludge, create a setting where the biomass may react with the wastewater, resulting in the degradation of pollutants into innocuous by-products (Ahmed and Lan 2012).

Attached-growth: Microorganisms are grown onto a solid surface in a fixed-bed reactor as part of a biological wastewater treatment technique known as attached growth (Mojiri et al. 2013). Microorganisms form a biofilm as wastewater runs over the surface of a medium, such as rocks, gravel, or plastic. Organic materials and contaminants in wastewater are broken down by a microbial population that calls the biofilm home. The bacteria in the biofilm metabolise the organic components in the wastewater as it flows through. Getting rid of organic material and nutrients like nitrogen and phosphorus from wastewater is a speciality of the attached growth method (Aziz et al. 2011). It also has benefits such as being durable, resistant to shock loads, and adaptable to different flow rates. Trickling filters and rotating biological contactors (RBCs) are two examples of attached-growth systems that play an essential role in wastewater treatment by removing pollutants and sustainably protecting water supplies. Furthermore, nitrification is lower, influenced by the low temperatures (Aziz et al. 2011), than that by suspended-growth systems and also by inhibition because of the high nitrogen content (Abdel-Shafy et al. 2023).

Aerobic treatment: Aerobic biological treatments are a type of wastewater treatment that uses oxygen-dependent bacteria to decompose organic materials and contaminants. These techniques are widely utilised in municipal and industrial wastewater treatment plants because they effectively remove organic compounds, nutrients, and other impurities. The efficient and highly effective technique employed for the treatment of landfill leachate involves the utilization of the traditional biological process, specifically the aerobic process. This process facilitates the elimination of biodegradable organic pollutants while simultaneously converting ammoniacal nitrogen into nitrite through the process of nitrification. Consequently, this results in the degradation of organic material in a highly efficient manner, thus enabling the convenient removal of nitrogenous compounds. It is important to note that this approach has been extensively studied and researched by Ilmasari et al. (2022) as well as Luo et al. (2020), who have provided valuable insights into its effectiveness and potential applications. Aerobic biological processes rely on suspended growing biomass in conventional activated sludge processes, sequencing batch reactors (SBRs), and aerated lagoons (Aziz et al. 2011). There are several varieties of attached-growth systems, such as the bio-filters and the moving-bed biofilm reactor (MBBR). Aerobic treatment can help remove the biodegradable organic contaminants and move the ammonium-nitrogen nitrification process along. The membrane bioreactor, which combines membrane separation with aerobic bioreactors, has recently garnered much interest (Ahmed and Lan 2012; Abdel-Shafy et al. 2023).

Activated sludge: Activated sludge is a popular and efficient biological wastewater treatment method that uses a microbial suspension in a liquid media to decompose organic matter and contaminants in wastewater. Wastewater is treated with this technique by combining it with a colony of microorganisms called activated sludge in aeration tanks. Bacteria and other microbes break down organic chemicals into harmless by-products like carbon dioxide and water (Kamaruddin et al. 2017). What is left of the mixture after the aeration phase settles in secondary clarifiers? This is the sludge that is returned to the aeration tank and used to keep the microbial population stable. Effluent treated in this way is more likely to be free of organic material, nutrients, and pollutants, making it compliant with discharge or reuse regulations. The flexibility, efficiency, and resilience of activated sludge make it an indispensable component of worldwide municipal and industrial wastewater treatment facilities. The treatment of leachate has been explored through the implementation of the Activated Sludge Process (ASP), which has showcased its viability by effectively removing organic carbon, ammonia, inorganic nutrients, and phenolic compounds (Ilmasari et al. 2022). However, it has been proven inadequate for treating landfill leachate (Abdel-Shafy et al. 2014). Despite being beneficial in the removal of nutrients, organic carbon, and ammonia contents, however, there are some other difficulties to consider: Municipal landfill leachate, anaerobically pre-treated, was nitrified at different temperatures (5–10°C) in an activated sludge reactor and the presence of plastic carrier material (Kamaruddin et al. 2017; Abdel-Shafy et al. 2023). Some of the disadvantages associated with this approach include the necessity for longer aeration periods, lasting approximately 20 h, and the inadequate capability of the sludge to settle properly (Wanner et al. 2014). Additionally, there is an overproduction of sludge and a high energy demand (Pant et al. 2010). The growth of microbes is hindered due to the elevated nitrogen content found in most leachates. Furthermore, the resulting sludge fails to meet established standards, necessitating an additional treatment technique to ensure compliance (Ilmasari et al. 2022). Aerobic post-treatment yielded an effluent of 150–500 mg COD L^−1^, lesser than 7 mg BOD L^−1^and lesser than 13 mg L^−1^.

Aerated lagoons: ‘Lagooning’ stops biomass growth. Aerated lagoons like the one in are cost-effective for removing organic and inorganic matter, as well as pathogens, in wastewater treatment, especially in developing countries where specialized skills are scarce (Frascari et al. 2004). Aerated lagoons, vast basins that use biological oxidation with a constant air supply, are a cost-effective way to remove microbial and organic load. Operators and decision-makers in developing countries choose these basins to treat landfill leachate because of their efficient operation and low maintenance and operational costs (Maia et al. 2015). Landfill-diluted leachate treated with artificial wetlands and anaerobic-aerobic lagoons removed over 70% N, P, and Fe (Ahmed and Lan 2012). Frascari et al. (2004) found that lagooning phenolic and organic debris reduced COD and phenol concentrations by 55–64% and 80–88%, respectively. Due to its reliance on microbial activity, this technique is susceptible to temperature changes. Temperatures below 15–20°C inhibit bacterial growth, slowing therapy (Abbas et al. 2009). Treatment effectiveness also depends on retention length. This is because it controls microorganisms’ lagoon stays and organic matter breakdown Frascari et al. (2004). Temperature greatly affects microbial activity, limiting lagoons.

Membrane bioreactors (MBRs): MBRs are advanced wastewater treatment systems that combine biological treatment with membrane filtration. They are widely used in various industrial and municipal applications to treat wastewater efficiently and produce high-quality effluent. Membrane technology combines the use of ASP and membrane units for efficient treatment, mainly applied to highly loaded leachates that are difficult to treat using other technologies (Gu et al. 2023). Moreover, membrane technology has proved to be efficient for the treatment of mature landfill leachates with recalcitrant pollutants (Zhang et al. 2020) and offers a stable process with low sludge production (Iorhemen et al. 2016a, b). MBRs provide an effective means of removing organic pollutants, suspended solids, and pathogens from wastewater. In addition, the membrane bioreactor (MBR) is a system that combines a bioreactor with membrane separation technology to provide a small footprint while producing high-quality effluent (Ahmed and Lan 2012; Teng et al. 2021). Biodegradation, adsorption, and membrane filtration are all combined in the Ultrafiltration-biologically active carbon (UF-BAC) hybrid membrane bioreactor system (Mansoorian et al. 2020). Overall, the procedure was between 95 and 98% effective at reducing organic carbon.

On top of that, in contrast to standard practices, the organisms responsible for the gradual degradation of biodegradable materials, such as nitrifiers, are probably not rinsed out of the process (Lu et al. 2021). Nevertheless, the most challenging aspect of membrane technology is the fouling occurrence due to the use of high organic strength leachate, excessive formation of biosolids, or excessive microbial growth, among other reasons. These challenging aspects substantially increase the operating cost (Abuabdou et al. 2020; Remmas et al. 2018).

Constructed wetlands (CWs): Constructed wetlands (CWs) mirror natural wetland ecosystems by incorporating essential features that clean leachates and wastewater of hazardous chemicals. Hydric soils with various microorganisms, aquatic flora, and a sand and gravel filtration system are essential (Stottmeister et al. 2003; Wdowczyk et al. 2022). Synthetic ecosystems filter and reuse urban, industrial, and agricultural water (Wu et al. 2014; Zhang et al. 2020). A built wetland can also remediate leachate biologically. Plants, media, and microbes are purposely included. Long, stringy-rooted water plants clean leachate well. *Scirpus validus*, *Limnocharis flava*, and *Ipomoaea aquatica* eliminate ammonia nitrogen. CW systems can recover biodegradable organic carbon and ammonia from landfill leachate (Dan et al. 2017). There are numerous ways to reduce nitrogen pollution, according to Mojiri et al. (2016). Adsorption on substrates, plant root absorption, ammonia volatilisation, biological breakdown, and biochemical translation into N2 (Badejo et al. 2020; Zhang et al. 2020). Free-water surface flow CWs flow on basin surfaces, while subsurface flow CWs flow below them. Based on subsurface water flow, the latter might be vertical or horizontal. Vertical subsurface flow CWs are utilized more than horizontal ones. Selecting vegetation in built wetlands (CWs) is crucial to leachate treatment. Their vital function in absorbing nutrients and heavy metals affects removal efforts (Klomjek and Nitisoravut 2005; Mbuligwe 2005). Thus, the inlet’s organic material and pollutants, temperature, and hydrology must be considered before selecting CW vegetation. Cost reduction, habitat creation, and aesthetics are all benefits of adopting CWs. They require a lot of space and may be weather-sensitive. CWs handle leachate sustainably and ecologically, improving water quality and the environment.

Moving beds biofilm reactors (MBBR): MBBR is a method of treating wastewater that uses biofilm technology to eliminate organic debris, nutrients, and pollutants. High-surface-area media carriers made of plastic are used in MBBR systems. These transport mediums are ideal for microbial biofilm formation (Saxena 2022). The media carriers are free to move around as the wastewater flows through the reactor, producing a dynamic environment in which the biofilm is continually exposed to clean water. This motion brings biofilm microbes into more direct and constant contact with organic and nutritional contaminants in the wastewater. A biofilm of the active biomass forms on the surfaces while porous polymer-carriers are hung and move continuously inside the aeration tank. In addition to rejecting organic matter and large quantities of ammonia in a single cycle, the process has several other benefits, including increased biomass production and shorter sludge-settling times (Abdel-Shafy et al. 2021). High ammonia concentrations did not prevent nitrification (Aziz et al. 2011). In contrast, it has been reported that granular activated carbon (GAC) can function as a porous surface adsorbing organic matter and furnish acceptable conditions for enhanced biodegradation (Aziz et al.^ 2011^; Abdel-Shafy et al. 2023), so a steady equilibrium can be achieved between the processes of adsorption and biodegradation. A very effective biological AC fluidised bed might remove as much as 70% of the refractory organics present. Ammonia levels were observed to be reduced by 85–87%, and COD levels by 61–82% (Aziz et al. 2011; Abdel-Shafy et al. 2023)

Sequencing batch reactor (SBR): Batch-operated activated sludge Sequencing Batch Reactors (SBRs) treat leachate and other effluent. Dynamic, flexible, suspended-growth biological therapy (SBR) technology has no steady-state condition. This process uses fill and decant-ASP with or without a clarifier. Al-Rekabi et al. (2007) and Mahvi et al. (2008) characterize the traditional SBR process as fill, react, settle, draw, and idle. Intermittently aerating the system allows all metabolic processes and solids-liquid separation to occur in a single tank, carefully regulated by a timed sequence (Alattabi et al. 2017). Duan et al. (2020) noted that the conventional SBR method uses a single reactor, leachate, aeration, settling, decanting, and waste collection to nitrify, denitrify, and phosphorous simultaneously. Leachate is effectively filtered of organic pollutants and suspended particles, improving operational adaptability and environmental impact. SBR also removes nutrients as well. SBR requires constant sludge control. According to Deng et al. (2018), aerobic leachate treatment in SBR can remove 75% COD and 99% -N in 20–40 days. Sequential anaerobic-aerobic reactor batch reactors remove 62% COD, 31% -N, and 19% with solid capture and less organics after 21 h. By efficiently abating organic waste, methanogenesis and denitrification may enhance early landfill nitrification (Deng et al. 2018). High-rate reactors shorten digestion time.

Phytoremediation system: Phytoremediation removes pollutants from soil and water using plants. A sustainable, cost-effective, and environmentally friendly repair approach (Kafle et al. 2022). Plants remove pollutants from landfill leachate by phytoextraction and phytovolatilization. Plants that accumulate heavy metals in their tissues are used for phytoextraction from leachate. Metals are collected from harvested plants. However, phytovolatilization uses plants that release pollutant gases. This method removes volatile pollutants from landfill effluent. While eliminating toxins, phytoremediation can also lower landfill discharge volume. Leachate water and nutrients help plants develop (Kafle et al. 2022). In landfill leachate treatment, phytoremediation is promising. It removes various pollutants from leachate in a sustainable, cost-effective, and ecologically friendly manner. Like CWs, phytoremediation depends on plant type. Abbas et al. (2009) used aquatic plants to remove COD, BOD, and heavy metals from landfill leachate with little migration from roots to aboveground portions. Lavagnolo et al. (2016) observed that leachate-irrigated oleaginous plants exhibited higher plant mass and COD, total nitrogen, and total phosphorous removal efficiency than control systems, with soil type also affecting plant growth. Phytoremediation reduces landfill leachate levels by evapotranspiration and recovers water and nutrients (Lavagnolo et al. 2016; Nagendran et al. 2006). Ponding systems: Ponding systems are an efficient and straightforward technique for treating landfill leachate (Adhikari and Fedler 2020). They rely on natural processes such as sedimentation, flocculation, and biodegradation to remove pollutants from leachate. The leachate is initially poured into a large pond, where it settles, then the denser solids sink to the bottom of the pond while the lighter solids flocculate and float to the surface and the settled solids are extracted from the reservoir, while the flocculated solids are skimmed off the surface (Adhikari and Fedler 2020). The clarified leachate is then circulated through an array of aerobic and anaerobic basins. In aerobic ponds, microorganisms utilize oxygen to decompose the organic matter in effluent.

Rotating Biological contactors: A Rotating Biological contactor (RBC) is a secondary treatment method where rotating disks with fixed media filters are used to remove organic material and ammonia from wastewater and leachates by submerging the disks partially in the wastewater and allowing their rotation to degrade the organic material, while specific microorganisms grow on the disk's surface and are later removed along with excess sludge (Miao et al. 2019). RBCs are well-suited for purifying landfill leachate, which is a highly concentrated wastewater that may contain high levels of organic matter, ammonia, and heavy metals. The treatment efficiencies of RBC systems for leachate treatment have varied across different studies due to different operational conditions; Wang et al. (2021) found complete ammonia oxidation but low COD removal of 38% when treating high-strength ammonia leachate with low BOD using RBCs with low substrate loading rates to promote nitrification. RBCs have a number of advantages over other forms of biological wastewater treatment systems for the treatment of landfill leachate. They are comparatively compact and simple to install in confined spaces. They are also exceptionally effective at removing organic matter and ammonia from effluent. Moreover, RBCs are comparatively simple to operate and maintain. RBC systems for leachate treatment, which have been extensively researched and analyzed by various studies, including Maheepala et al. (2022), have proven to possess an exceptional ability in nitrification. The effectiveness of these systems in removing nitrogen compounds from wastewater has been well-documented. However, like any technology, RBC systems come with their limitations, and the primary drawback lies in their vulnerability to extreme weather conditions. In order to maintain optimal performance and prevent any potential damage, these systems must be adequately shielded from excessive sunlight, wind, rain, and even snow, as highlighted by Wang et al. (2021) in their research. The need for protection against these environmental factors is crucial to ensure the longevity and efficiency of RBC systems in wastewater treatment applications.

Anaerobic treatment of leachate*:* The oldest wastewater treatment method is the anaerobic process, from the late nineteenth century. This therapy uses microbes to convert organic materials. Anaerobic organisms live without molecular oxygen using nitrogen, phosphorus, sulfur, potassium, calcium, and magnesium; these anaerobes can promote microorganism development. Anaerobes may acidogenize and methanogenize leachate (Tawfik and ElBatrawy 2012). Organic trash and pollutants in landfill leachate are broken down by anaerobic microorganisms without oxygen. Anaerobic digestion produces biogas and carbon dioxide from microbes, simplifying leachate organic components (Deng et al. 2018). In a closed reactor or digester, leachate is fed to anaerobic bacteria. Anaerobic treatment reduces organic load, eliminates odours, and generates energy-generating biogas (Maillacheruvu and Fayyaz 2007). The treated leachate may need aerobic or physicochemical treatment before discharge. Anaerobic landfill leachate treatment can generate renewable energy and reduce greenhouse gas emissions. Anaerobic digestion produces fewer solids and conserves energy due to its delayed reaction rates (Maia et al. 2015). With CH_4_, the digester may be heated to a pleasant 35°C.

Anaerobic ammonium oxidation (Anammox): The process of anaerobic ammonium oxidation (Anammox) transforms ammonium (${NH}_4^{+}$) to dinitrogen (N2) gas in anoxic environments (Strous et al. 1999). A consortia of bacteria oxidizes ammonium without oxygen. Anammox is a novel landfill leachate treatment that shows potential. Organic debris, ammonium, and other contaminants are concentrated in landfill leachate. Leachate with high ammonium levels can cause eutrophication and other water quality concerns. Anammox removes ammonium from leachate efficiently and sustainably (Heijnen et al. 1998). Anammox is usually done in a reactor at 20–30°C and 7–8 pH (Strous et al. 1999). After seeding the reactor with Anammox bacteria, leachate is pumped in. Leachate ammonium provides electrons for bacteria, which generate dinitrogen gas. Anammox removes ammonium up to 90% efficiently. This yields far greater removal efficiencies than aerobic oxidation or chemical precipitation (Hu and Deng 2011). Anammox’s energy efficiency makes landfill leachate treatment cost-effective. Several variables can alter Anammox’s performance. These include leachate pH, content, and temperature. Organic substances can also inhibit Anammox (Hu and Deng 2011). These elements may be modified to optimize the Anammox process.

Nitrification and denitrification: Nitrification and denitrification are frequently combined in landfill leachate treatment in a two-step process known as “nitrogen removal” or “nitrification-denitrification.” Alternating aerobic and anoxic conditions are provided to maintain both nitrifying and denitrifying microorganisms. Nitrification takes place during the aerobic phase as ammonia is transformed into nitrate. Denitrification happens in the anoxic phase as nitrate is converted to nitrogen gas. This two-step procedure successfully eliminates nitrogen compounds from leachate, decreasing its environmental impact and allowing it to be safely discharged or reused. To achieve optimal performance of the bacteria participating in the nitrification and denitrification processes in landfill leachate treatment, operational variables such as pH, dissolved oxygen levels, and organic carbon availability must be carefully controlled.

Furthermore, enough mixing and aeration are required to keep the required aerobic and anoxic conditions inside the treatment system. Nitrification and denitrification, when properly built and managed, serve a critical role in minimising the nitrogen-related environmental hazards connected with landfill leachate wastewater. Nitrification and denitrification are critical stages in lowering ammonia and nitrate concentrations prior to discharge or reuse of treated leachate (Zhang et al. 2020).

Anaerobic digestion: Leachate treatment often employs the biological process of anaerobic digestion, which includes the degradation of organic materials by anaerobic microbes in the absence of oxygen. The organic load and hazardous chemicals in leachate may be greatly reduced by anaerobic digestion, a procedure that is both efficient and ecologically benign. The organic chemicals in leachate are decomposed by bacteria during the anaerobic digestion process into molecules like methane, carbon dioxide, and water (Strous et al. 1999). The utilization of digesters or bioreactors, which provide a closed, oxygen-free environment, is commonplace during this microbial activity. Biogas, which is comprised primarily of methane, is produced as a byproduct of anaerobic digestion and may be collected and used as a sustainable energy source (Kumar et al. 2021). There are several upsides to adopting anaerobic digestion for leachate cleanup. First, it aids in reducing the negative effects of leachate on the environment by keeping potentially hazardous contaminants from seeping into the ground and water. Second, producing biogas as an alternative energy source helps the environment and cuts down on fossil fuel use, both of which are important for long-term sustainability. The resource efficiency is increased since the treated leachate may be safely released or reused. However, the composition of the leachate, temperature, pH, and retention duration in the digester are only a few of the elements that might affect the efficacy of anaerobic digestion in leachate treatment. To increase microbial activity and waste elimination, ideal conditions must be preserved.

Anaerobic filters: Anaerobic filtration is a prominent leachate treatment. Anaerobic digestion involves microorganisms breaking down organic materials without oxygen. Anaerobic filters clean landfill leachate well due to their high concentrations of organic pollutants and other impurities (Weiland 1987). Leachate-treating anaerobic filters employ a packed-bed reactor containing pebbles, plastic, or other support materials. Anaerobic microorganisms degrade leachate organic molecules on the medium’s surface. The reactor’s biofilm of anaerobic bacteria digests organic waste as leachate runs through, creating methane, carbon dioxide, and water. This biological process reduces organic load and removes harmful compounds from leachate. Anaerobic filters can handle leachate’s strong and complex organic waste, making them ideal for treatment. The reactor’s medium keeps the anaerobic microbial community stable, ensuring effective treatment independent of leachate composition (Pal et al. 2010). Kumar et al. (2021) used an up-flow anaerobic filter to remove organic materials from landfill leachates. The filter considerably reduced COD in landfill leachate, with biogas generation and methane levels within defined parameters. Anaerobic filters also use less electricity and are easier to maintain. They also produce biogas, which may be harvested and utilized as renewable energy, reducing greenhouse gas emissions. Anaerobic filters, like every therapeutic device, have limits. Maintenance and monitoring may be needed to maintain microbial activity and discover and correct blockages before they cause issues. Temperature, pH, and leachate contaminants can also impact method efficacy.

Anaerobic sequencing batch reactors: Anaerobic sequencing batch reactors (ASBRs) can treat leachate. The ASBR works like the aerobic sequencing batch reactor but without aeration. Batch-operated ASBRs are multi-stage treatment systems. They have garnered recognition for their ability to handle difficult organic waste like landfill leachate. Because they recycle leachate through numerous phases in the same reactor, ASBRs handle it well (Jiraprasertwong et al. 2018). Therapy plans usually have four phases: The reactor is filled with leachate to a target volume during filling. In the react phase, reactor anaerobic bacteria degrade leachate’s organic components, creating biogas (mainly methane) and stable organic compounds.

Let the treated leachate settle and separate particles after the react phase when biogas generation diminishes. Decanting the filtered effluent leaves solids at the reactor’s base (Jiraprasertwong et al. 2018). Timur and Ozturk (1999) found that a lab-scale ASBR could treat municipal landfill leachate, removing 64 to 85% of COD depending on loading rates and converting 83% of COD into biomethane. The batch operation gives ASBRs leachate treatment advantages. Process control and leachate property adaptation are improved. ASBRs are also more resistant to influent flow and composition, making them effective for leachate with different organic loads (Yong et al. 2018). Another benefit of ASBRs is organic waste diversion and biogas production. Leachate treatment biogas may be utilized as renewable energy, boosting the operation's long-term profitability. ASBRs have severe limitations, but so does any technology (Yong et al. 2018). Due to its intricacy, the system may require more advanced operation and maintenance than simpler treatment systems. Inhibitory chemicals or dangerous substances in leachate, which anaerobic bacteria would normally thrive on, may also hamper treatment.

Hybrid filters: Hybrid filters are a type of leachate treatment that combines many treatment methods into a single system to improve overall performance. Because of the complex nature of landfill leachate, innovative hybrid solutions have been developed to handle it (Kececioglu et al. 2016). Hybrid filters are a method of treating a problem by combining multiple treatment methods, usually in a sequential or parallel fashion. The synergistic benefits of each process are amplified when several technologies are used in tandem, leading to more efficient pollution reduction and a greater treatment level than would be possible with any technology used alone. Hybrid filters like this are often used in leachate treatment (Kececioglu et al. 2016). A hybrid of biological (such as anaerobic or aerobic digestion) and physical (such as filtration or sedimentation) treatment procedures. This method can purge leachate of both organic and inorganic contaminants efficiently.

**Thermal treatment processes:** Due to the pervasive and difficult-to-treat nature of certain of the contaminants found in landfill leachate, thermal treatment techniques play a vital role in leachate treatment (Wang et al. 2022). These procedures make use of heat to hasten several chemical and physical reactions, ultimately resulting in the breakdown, volatilization, or destruction of organic and inorganic pollutants in the leachate. When treating leachate, thermal treatment methods such as:

1. Leachate can be incinerated in an oxygen-rich atmosphere by a controlled burning process called incineration. Carbon dioxide and water vapour are produced from organic molecules as the heat does its work, while inorganic substances become ash. To remediate high-strength leachate and lessen the amount of trash that has to be disposed of, incineration is a viable option. However, it needs to be closely monitored and managed so that dangerous air pollutants aren't produced (Wang et al. 2022).
2. Thermal treatment and oxidation in one step using high-pressure steam characterize the process known as wet air oxidation (WAO). Organic pollutants in leachate are degraded by heating in the presence of oxygen. When opposed to incineration, WAO has less air emissions and can efficiently remove refractory organic contaminants (Schnell et al. 2020).
3. High-temperature anaerobic digestion (HTAD) is a method of decomposing organic materials by microorganisms that involves heating leachate to higher temperatures than is typical for anaerobic digestion. Faster digestion and biogas generation can be achieved due to the increased activity of anaerobic microbes as a result of the higher temperature (Candelier et al. 2016).
4. Through a process called pyrolysis, organic compounds in leachate are broken down into their parts—char, liquid oils, and gaseous products like syngas and volatiles—when the liquid is heated to high enough temperatures without the presence of oxygen. Energy recovery and trash minimization are two of the many applications of pyrolysis.
5. When organic components in leachate undergo gasification, a thermal process, the resulting gases are a combination of carbon monoxide, hydrogen, and methane. Synthesis gas (syngas) is the name given to the byproduct gas, which has potential as a clean energy source.
6. Thermal treatment technologies are helpful for dealing with stubborn and complicated contaminants that are difficult to remove using more conventional biological or physical approaches. They offer benefits, but they also have problems, including high energy demands and possible air pollutants. Effective emission control and waste disposal are just two aspects of process management that are crucial to the long-term viability of these operations (Candelier et al. 2016).

Up-flow anaerobic sludge blanket (UASB)*:* Process development and high-rate treatment technologies have advanced greatly with the UASB module. A unique design allows the UASB to separate liquid, gas, and solid phases in one container. UASB reactors are being explored for leachate treatment due to their energy efficiency, user-friendliness, and low sludge generation. Methane and hydrogen are created during operations and can be used as fuel. Pre-treatment of landfill leachate to remove particulates may increase reactor performance. Post-treatment with the UASB reactor is necessary to meet discharge requirements (Tawfik et al. 2012). Volumetric organic loading ratios between 20 and 35 benefit from this method’s better treatment and shorter hydraulic retention duration. At 20–23° Celsius, COD was decreased by over 70%, and at 35° Celsius, by over 80%. COD was reduced by 92% due to low to moderate organic loading ratios (Kurniawan et al. 2006). Maintaining a temperature between 15 and 35° Celsius before high-rate treatment reduces heat consumption (Miao et al. 2019). Anaerobic lab-scale suspended-growth digesters removed 80–90% and 55% of COD at 35°C and ambient temperature (Noerfitriyani et al. 2018). The extraordinary results shown by UASB suggest that a high-rate treatment at lower temperatures might minimize leachate heating, presenting an exciting and economically viable option. Note that this treatment approach is prone to harmful substances, including ammonia and heavy metals (Timur Özturk 1997). Noxious substances are not allowed (Kuusik et al. 2014).

Fluidised bed reactor: A fluidised bed reactor (FBR) is a reactor utilised in various industrial processes and applications, such as chemical reactions, catalytic processes, and wastewater treatment. Solid particles floating in a fluid (typically a gas or liquid) behave like a fluidised mass in a fluidised bed reactor. This happens when there is enough upward movement of fluid, causing the solid particles to become buoyant and appear to be boiling. It has been demonstrated that combining biodegradation and adsorption techniques enables excellent removal of different organic chemicals (Castilhos et al. 2009). The biological AC fluidised bed was discovered to be far more effective than conventional methods, such as fixed film and activated sludge, in treating leachate from prehistoric landfills.

#### Chemical and physical treatment

Chemical and physical processes are often used to remove different pollutants and toxins from landfill leachate efficiently. The objective is to minimise the adverse effects of waste disposal on the surrounding environment while producing effluent of the appropriate quality for safe discharge or potential reuse. The processes include the reduction of toxic compounds, floating material, colloidal particles, suspended solids and colour via chemical oxidation, adsorption, flotation, air stripping or coagulation/flocculation. Chemical and physical approaches to treating landfill leachate are summarised here (Abdel-Shafy et al. 2023).

Floatation: Dissolved air flotation (DAF) physically removes suspended sediments and other tiny pollutants from wastewater. It is utilized in municipal and industrial wastewater treatment because it filters out non-settling particles. As air or gas enters the wastewater system under pressure, small bubbles adhere to particles and raise them to the top for removal. The sludge or foam is skimmed off and removed from the wastewater system. Flotation removes macromolecules, germs, fibres, colloids, humic acids, and ions from solutions, according to several studies. Combining FeCl3 coagulation with DAF was tested for treating semi-aerobic liquid-landfill leachate. RSM and CCD helped the researchers identify optimal values for all variables. All turbidity, colour, chemical oxygen requirement, and ammonia nitrogen (-N) were eliminated to maximum values. We achieved 50% turbidity reduction, 75% COD removal, 93% colour retention, and 41% (-N) retention. Overall, flow rate and pressure removed fewer pollutants. The DAF system under study’s performance and efficiency depend on these two components. These novel discoveries have already been included in commercial DAF landfill leachate treatment (Mohd et al. 2011). Post-treatment with column flotation extracted residual humic acids and non-biodegradable compounds from simulated landfill leachate (Abdel-Shafy et al. 2020). Ideally, 60% of humic acids could be removed (Dabaghian et al. 2018).

Chemical Coagulation–flocculation: The use of chemical coagulants and then flocculants to destabilise and aggregate suspended particles and colloidal debris in leachate wastewater is the chemical coagulation-flocculation process, and it is commonly used for treating wastewater. This method successfully eliminates small particles, organic compounds, and certain dissolved pollutants, making it a vital stage in water and wastewater treatment facilities. Chemical coagulation and flocculation are extensively utilised in leachate wastewater treatment and the treatment of old and stabilised landfill leachates (Assou et al. 2016). It has been used successfully as a pre-treatment to remove non-biodegradable organic contaminants or before the reverse osmosis phase. Coagulants commonly employed include ferrous sulfate, ferric chloride sulfate, ferric chloride, lime, and aluminium sulfate (Ghafari et al. 2009). It was discovered that bio-flocculants are a feasible alternative to typical inorganic coagulants, with a dose of 20 mg L^−1^ eliminating more than 85% of humic acid and 90% of heavy metals (Abdel-Shafy et al. 2015; Abdel-Shafy et al. 2023). Process optimisation was investigated, including assessing the pH effect and selecting the most appropriate coagulant (Abdel-Shafy et al. 2015). and the best experimental circumstances. Iron salts offered sufficient COD reductions of up to 5%, whereas aluminium salts or lime gave moderate comparable values ranging between 10 and 40% (Mojiri et al. 2013). COD reduction of up to 50% might be accomplished by increasing the floc-settling rate with either a coagulant combination or the coexistence of flocculants and coagulants (Aziz et al. 2011). Nonetheless, several drawbacks may be observed: constant sludge volume generation and increased aluminium or iron content in the liquid phase (Mojiri et al. 2013).

Chemical precipitation: Chemical precipitation turns dissolved contaminants into insoluble forms in leachate wastewater by adding chemicals. By sedimenting or filtering the water, contaminants may be readily removed. With chemical coagulants and aids, this treatment removes and co-precipitates pollutants from landfill leachate and wastewater. Abdel-Shafy et al. (2015) precipitated heavy metals from wastewater at different pH values. Scientists studied Ni, Cu, and Mn concentrations chemically and physically. Using NaOH at pH 9.5, Ni, Cu, and Mn were removed at 96.0, 97.5, and 90.0%. The study used 50 mg/L sodium hydroxide and ferric chloride and a variety of pH levels. Every element examined was eliminated above 98% efficiently at pH 12.0. When applied with NaOH, 70 mg/L alum removed 100% Ni and Cu and 84% Mn at pH 12.0. Testing different lime (CaO) concentrations showed that 11.0 was the best pH for Ni, Cu, and Mn removal. The effects of limestone (CaCO_3) concentrations were studied further. A one-unit limestone pH increase from 2.0 to 5.85 removed 90.2%, 100%, and 75.1% Ni, Cu, and Mn, respectively. After increasing CaCO3 to 3.0 g/l, Ni and Cu were removed at rates more than 100%, whereas Mn was removed at 90.6% (Ghafari et al. 2009). The solubility product (SP) is critical to pollutant precipitation, especially metals. Since effluent laws need a pH between 6 and 9, carbonate is recommended as a treatment option. Compared to other precipitation processes, lime is cost-effective. Lime is used to treat wastewater. However, calibrating the pH is tricky. Acid can be added as needed to fix this (Ghafari et al. 2009). In pre-treatment, this technique reduces pollutant potency (Ghafari et al. 2009).

Adsorption treatment: Adsorption transfers organic compounds from liquid to solid surfaces. Adsorption is mass transfer. Water molecules adsorb onto materials having a large interior surface area. The adsorbent is the surface for adsorption, while adsorbate is the substance being adsorbed. One of the most successful post-treatment approaches for removing ${NH}_4^{+}-N$ from landfill leachate is adsorption (Jiang et al. 2019). Adsorption is simpler and cheaper to set up. For landfill leachate treatment, activated carbon (AC) and biological treatment have been used (Abdel-Shafy et al. 2021). No matter the starting organic matter content, this approach may reduce COD more than chemical treatments (Aziz et al. 2011). Continuous carbon column regeneration is the biggest disadvantage. Another option is powdered AC in huge volumes. Biologically treated landfill leachate may contain less inert COD, non-biodegradable organics, and colour. According to Aziz et al. (2011), AC exhibited the highest adsorption capacity for non-biodegradable organic chemicals, reducing COD by 85% and leaving 200 mg L^−1^. Air stripping and ammonium coagulation-flocculation were followed by biological treatment in an aeration tank with 2 g L^−1^ powdered AC and zeolite as adsorbents in repeated fed-batch mode to remove nearly 87% and 77% of COD, respectively. Metal concentrations might be decreased by filtering water with granular carbon before standard treatment. Limestone also removes metals from leachate wastewater (Baun et al. 2004). Adsorbents with a limited surface area, macroporous structure, surface inactivity, and instability have been used to treat landfill leachate, which cannot be obtained from commercial or natural sources such as zeolites, clay minerals, and biopolymers. Drawbacks include adsorbent regeneration and excessive absorbent usage.

Chemical oxidation: Water treatment with powerful oxidizing agents breaks down organic and inorganic pollutants. Breaking down complicated compounds into their basic elements lessens their toxicity. Industrial and municipal wastewater treatment uses chemical oxidation to destroy persistent organic pollutants and enhance water quality. Chemically oxidizing stale or well-stabilized leachate converts organic molecules into water and carbon dioxide, completing mineralization. Improving recalcitrant organic pollutants' biodegradability helps future biological treatment become cheaper. Powerful oxidants include O_3 and H_2 O_2, ultrasonic (US), ultraviolet (UV), electron beam (EB) irradiation, photocatalysts, and transition metal ions. Using oxidants or Fenton oxidation and magnetic ionic exchange resin through 2D-CoSEC can improve treatment efficiency on stabilized leachates, where only 30% of COD is removed after 1 h of ozonation at 1.3–1.5 gO3/g COD (Qi et al. 2019). Qi et al. (2019) say the process removes 90% of organic waste. Qi et al. (2019) found that the a/UV approach increased the BOD5/COD ratio from 0.1 to 0.45.

Fenton process: The Fenton process is an advanced oxidation method used for treating wastewater and degrading organic and inorganic pollutants. It is based on the generation of highly reactive hydroxyl radicals (OH) through the reaction between hydrogen peroxide (H_2_O_2_) and ferrous iron (Fe^2+^). The biodegradability was enhanced using the Fenton procedure, and following oxidation, BOD_5_/COD ratios of around 0.5 were found (Aftab et al. 2018). Electron-beam radiation or photocatalytic therapy to break down humic compounds has also been described as effective (Jokela et al. 2002). The high expense of therapy is a direct result of the radiators’ (ultrasounds, ozonisers, UV lights, etc.) heavy reliance on electrical power (Aftab et al. 2018). Furthermore, substantial oxidant dosages are required for the complete mineralisation of the contaminants, which is seen as economically costly. Because intermediate oxidation products can occasionally increase the leachate’s toxicity (Aftab et al. 2018). Since H_2_O_2_ and Fe^2+^ are both non-toxic and inexpensive, the Fenton method is the most cost-effective compromise. However, Fenton’s procedure necessitates pH adjustment to maintain a low (Aftab et al. 2018).

Air stripping: Air-stripping leachate wastewater removes VOCs and other dissolved gases. Based on mass transfer, water-volatile chemicals are released into the gas phase when air contacts them. VOCs are removed by air stripping in drinking water, industrial, and municipal wastewater treatment (Zhang et al. 2020). At little cost and with little equipment, air stripping may extract ammonium nitrogen -N from landfill leachates. Studies back this up. To maximize process efficiency, air stripping is done in a packed tower to remove and recover ammonia from wastewater (Zhang et al. 2020). Temperature, pH, piling length, and air-to-liquid ratio impact stripping performance (Provolo et al. 2017), increasing cost and salinity, which harms biological treatment. Jokela et al. (2002) found that 89% of ammonia is reduced at pH 11 and 20°C over 24 h retention period, proving that treatment with H2 or HCl and a high pH are necessary for efficiency. In stripping tanks, 309–368 mg L^−1^ ammonia-nitrogen was removed in 1 day (Jokela et al. 2002) despite an initial ammonia concentration of 0.5–0.7 gNL^−1^. Furthermore, 93% *NH*_4_ concentration was removed. The release of *NH*_4_ into the atmosphere might create air pollution unless HCl is able to absorb it. When lime is used to adjust pH, calcium carbonate scaling occurs in the stripping tower, which requires bigger towers.

Microfiltration: Microfiltration (MF) is a membrane filtration process used in wastewater treatment to remove suspended solids, large particles, and bacteria from water. It operates at low pressure, and its membranes have relatively large pore sizes, allowing water molecules to pass through while retaining larger contaminants. MF is effective in improving water quality, and it is commonly used as a pre-treatment step before other membrane processes or conventional treatment methods to prevent fouling and enhance overall efficiency. While it has limitations in removing dissolved substances and susceptibility to fouling, MF remains a versatile and economical solution for leachate wastewater treatment, contributing to water safety and environmental protection treatments (Magri et al. 2021; Zuo et al. 2018). RO, UF or NF, together with chemical treatments, are other types of (MF).

Ultrafiltration (UF): UF membrane separation removes suspended particles, colloids, macromolecules, and certain microbes from water and wastewater. Size exclusion allows UF to retain smaller particles and molecules since semi-permeable membranes have lower pore diameters than microfiltration. UF easily removes macromolecules and particles. According to tests, UF effectiveness depends on membrane material. Ultrafiltration technologies like the MBR filter prevent germs with 0.01–0.1 lm pores. Stacked membrane plates may scale the system quantitatively from tiny to large. To prevent RO membrane fouling, UF eliminates leachate’s higher molecular weight components. Organic material fractionation makes UF an appropriate RO pre-treatment step. The biological post-treatment of landfill leachate may also employ UF. Additionally, commercial membrane bioreactors using UF membranes handle leachate well (Zuo et al. 2018).

Nano-filtration (*NF*): NF removes divalent ions, organic debris, and certain micropollutants from water and wastewater. NF removes certain pollutants selectively with smaller pore diameters than ultrafiltration but bigger than reverse osmosis. The molecular cut-off size of NF polymeric films is 200–2000 Da. Sulfate ions and liquid organic substances are frequently rejected. Conversely, salt and chloride rejection is negligible (Amaral-Silva et al. 2016). Amaral-Silva et al. (2016) found that NF treatment of landfill leachates removes 60–70% COD and 50% ammonia at 3 m/s and 6–30 bar trans-membrane pressure. Physical methods and nano-filtration removed 70–80% of recalcitrant COD (Amaral-Silva et al. 2016). However, dissolved inorganic and organic detritus, colloidal, and suspended particles must be managed effectively to prevent membrane fouling. The first RO systems to remediate waste leachate employed spiral wounded and tubular modules in 1984. Over 98% of COD and 99% of heavy metal concentrations were rejected (Talalaj 2015). The disc-tube module (DT-module) developed intriguing new technology. For scaling, fouling, and biofouling removal, the open channel module works well. However, pressure-driven membranes have two major drawbacks: membrane fouling, which reduces lifespan and process productivity, requires pre-treatment or chemical cleaning. Additionally, the process generates a lot of concentrates that must be processed or discarded. High-pressure DT modules with 120 and 200 bar trans-membrane pressures are now available (Talalaj 2015; Wang et al. 2021).

Co-treatment of landfill leachate with domestic wastewater: Leachate wastewater is mixed with residential wastewater to reduce the concentration of organic compounds like ammonia. Biodegradability was increased, and the BOD_5_/COD ratio was balanced in landfill leachate treatment by mixing residential wastewater with leachate wastewater before treatment. (Mojiri et al. 2016; Zhang et al. 2020; Ishaq et al. 2023). Bio-electrochemical systems (BES) have recently gained a lot of attention because they allow microorganisms to be used as promotional agents or catalysts in the conversion of the chemical energy of the electron donors into electricity, bypassing the disadvantages of most of the other approaches (Ishaq et al. 2023).

Ion exchange and adsorption: Ion exchange reactions remove dissolved ions from solutions and replace them with similar-charged ions. A resin bed or ion exchanger exchanges ions between a fluid solution and a solid substance. This technique effectively removes ${NH}_4^{+}$ Ions with exceptional affinity. It is also simple, inexpensive, and eco-friendly. Ion exchange eliminates ammonia. Many ion exchangers and adsorbents, including zeolite, have been employed for years (Huang et al. 2018; Zhang et al. 2020). Zeolite is the most popular ion exchanger due to its high capacity and unique pore structure. Adsorption depends on pH, temperature, particle size, beginning ammonium concentration, contact time, and dosage. Ammonia adsorption is strongly affected by solution pH (Dong et al. 2019). A pH above 7.0 results in ${NH}_4^{+}$, which cannot be exchanged onto the adsorbent. At pH 5.0, *H*^+^ competes with adsorption sites, reducing removal. Huang et al. (2018) found that pH 5–8 is optimum. Zeolite and synthetic resins are commonly used to remove ammonium ions from wastewater (Prajapati 2014), but polymeric ion exchangers and hybrid cation/anion exchangers have also been studied. However, reagent regeneration, desorption during ion exchange, pH variations, and restricted reusability make this approach expensive (Adam et al. 2019).

Breakpoint chlorination: The widely used technique of breakpoint chlorination effectively transforms *NH*_3_ − *N* into *N*_2_, but it is primarily employed for fine-tuning wastewater rather than removing substantial amounts of nitrogen. This involves chlorinating water that contains ammonia, initially raising the residual chlorine (Abdolali et al. 2017). The combined chlorine and ammonia levels decrease together while the free chlorine increases, ultimately eliminating *NH*_3_ − *N* as *N*_2_. In the *NH*_3_ − *N* removal process, chlorine reacts with *NH*_3_ − *N* to form monochloramines, which then react with chlorine to produce dichloramine and eliminate *NH*_3_. Finally, free chlorine appears after the breakpoint, indicating the complete removal of *NH*_3_ − *N* , with a stoichiometric ratio of *Cl*_2_: *NH*_3_–N weight being 7.6:1. The procedure of adding enough chlorine or sodium hypochlorite to wastewater to convert the -N to N_2_ before discharging it into the atmosphere is called “breakpoint chlorination.” Wastewater’s free chlorine level is reduced to a point where only trace amounts of ammonia remain when *Cl*_2_ is added to the mixture (Dong et al. 2019; Ishaq et al. 2023). When extra *Cl*_2_ is supplied indefinitely, the concentration of free chlorine increases, and a breakpoint is formed. This approach is frequently used as an advanced treatment but is unsuitable for treating large amounts of wastewater with high --N levels. Zhang et al. (2020) and Ishaq et al. (2023) showed that combining UV irradiation at 254 nm with chlorination increased the ammonia removal rate and efficiency compared to breakpoint chlorination alone. The design of a breakpoint chlorination system is fairly simple, with the only requirement being a thorough mixing of chlorine with wastewater. The amount of *NH*_3_ − *N* and the level of treatment determine the size of the chlorine-producing and feed device. However, disadvantages include the need to remove chloramines if the breakpoint is not reached and reduced *NH*_3_ − *N* removal efficiency if chlorine-reducing compounds are present.

Internal micro-electrolysis: Leachate treatment utilizes internal micro-electrolysis, a cutting-edge physicochemical treatment method, to get rid of pollutants such as refractory organic compounds and heavy metals. In an electrochemical treatment reactor, reactive materials or electrodes are used to aid the breakdown and removal of contaminants through electrochemical processes (Chen et al. 2021). The following are the standard stages involved in internal micro-electrolysis:

1. Materials for the electrodes are selected for their capacity to catalyze the electrochemical processes necessary for the elimination of the pollutants of interest. Iron, aluminium, and other metal alloys are frequently used as electrodes.
2. The leachate is introduced to the treatment reactor's reactive materials or electrodes. Granules or particles are the most common shape that reactive materials take (Zhao et al. 2023).
3. Oxidation-reduction (redox) processes occur when leachate comes into contact with reactive materials. Electrodes made of iron corrode, producing electrons that can neutralize pollutants in the leachate (Zhao et al. 2023).

Organic pollutants, heavy metals, and other contaminants in the leachate may be broken down and removed thanks to the electrochemical processes triggered by the reactive materials. Reactive materials can oxidize organic chemicals or convert them to less hazardous forms, and heavy metals can be precipitated out of solution or adsorb onto their surfaces (Boonnorat et al. 2014).

Advanced oxidation process: Refractory organic pollutants and impurities may be removed from leachate utilizing innovative and strong treatment technologies called advanced oxidation processes (AOPs). To successfully oxidize and break down complex organic chemicals present in the leachate, AOPs generate extremely reactive hydroxyl radicals (OH) through numerous chemical processes (Chen et al. 2021).

#### Electro-chemical oxidation

The process of electrochemical oxidation is a modern and efficient approach to treating leachate. In this method, an electric current is used to initiate a series of electrochemical reactions that break down the organic and inorganic contaminants present in the leachate (Talebian et al. 2018). Popularity has increased for this technique because of its success in dealing with complicated contaminants that are resistant to conventional treatment procedures. An electrolytic cell with electrodes submerged in the leachate is used in electrochemical oxidation. Electric current passing across the electrodes sets off a chain reaction of oxidation and reduction, which decomposes organic materials and converts pollutants into less toxic byproducts (Talebian et al. 2018).

Leachate treatment relies heavily on electro-chemical reactions, including the following:

1. Electrolysis: It is at the electrodes that oxidation and reduction processes take place, making electrolysis the site of these chemical events. Organic molecules are easily degraded by reactive oxygen species (ROS) produced by anodes, such as hydroxyl radicals (OH) and peroxides (*H*_2_*O*_2_). The cathodes, meantime, aid in the elimination of some inorganic toxins (Luo et al. 2019).
2. Electro-Fenton process: As a subset of electro-chemical oxidation, the electro-Fenton process includes the creation of ferrous ions (*Fe*^2+^) from the dissolution of iron electrodes. Together with hydrogen peroxide, these ferrous ions generate even more reactive oxygen species (ROS), which accelerate the oxidation of organic pollutants (Luo et al. 2013).
3. Photoelectro-Fenton process: Combining ultraviolet (UV) light with the electrochemical oxidation process to activate the ROS and increase oxidation efficiency yields the photoelectro-Fenton process. The photoelectro-Fenton process, as this combination is called, is a powerful tool for the long-term decomposition of persistent pollutants. However, there are certain obstacles to think about, such as the possibility of electrode fouling, high running expenses owing to power consumption, and the requirement for rigorous monitoring and control to optimize the process(Umar et al. 2010).

Electro-coagulation: Leachate can be treated by electro-coagulation, a cutting-edge and efficient electrochemical water treatment technology (Bektaş et al. 2014). By passing an electric current through the leachate, pollutants in the water are coagulated and flocculated. This method is effective in cleaning leachate of contaminants such as suspended particles, metals, and organic material. Metal electrodes are submerged in the leachate, and an electric current is run between them to thicken the liquid, creating an electro-coagulation system. Metal ions, often aluminium or iron, are liberated from the electrodes as the current runs (Sabarudin and Kartohardjono 2020). These metal ions coagulate the leachate by reacting with water to produce hydroxide or oxide species. Leachate’s charged particles and pollutants are neutralized by the coagulants produced during electro-coagulation, which then cause them to cluster together into bigger aggregates known as flocs. These flocs are simple to filter out or allow to settle out. As a result, the amount of suspended particulates in the leachate is greatly diminished, and other colloidal and dissolved contaminants are eliminated.

Resin membrane filtration: Leachate can be cleaned via resin membrane filtration, a hybrid of ion exchange and membrane filtering. Porous resins can store contaminants (Conidi et al. 2015). Liquid contaminants attach to the resin membrane during filtration. Resin membrane filtering removes many contaminants from liquids. It effectively removes organic contaminants like leachate. Leachate, which escapes from landfills, contains salts, heavy metals, and organic waste. Leachate purification using resin membrane filtering is innovative and promising. Italian researchers cleaned waste leachate with resin membrane filtration in 2017. Resin membrane filtration can remove 99% of leachate organic material, according to studies (Conidi et al. 2015). Resin membrane filtration for leachate purification is novel. The technique efficiently removes most contaminants from liquids.

Reverse osmosis and forward osmosis: RO membrane filters. Semipermeable membrane filtration uses a thin membrane. Incredible, this membrane prevents particles and dissolved components in water or wastewater by a little pressure differential. These particles and components cannot enter influent effluent streams (Talalaj 2015). This process, unlike biological methods, separates contaminants into permeate and concentrate. Membrane methods filter solution components. By size, they divide components (Adam et al. 2019). Reverse or forward osmosis (RO or FO), membrane-based methods, treat rainwater that soaks through landfills or other waste storage areas (Wang et al. 2021). Reverse osmosis and semipermeable membranes filter leachate of dissolved solids, organic compounds, and other pollutants. Under pressure, a RO membrane eliminates most salts and pollutants from leachate. Reject or concentrate is concentrated effluent, whereas permeate is clean membrane water (Talalaj 2015). Forward osmosis draws liquids across a semipermeable membrane using osmotic pressure from leachate. No external pressure is needed because the fluids' osmotic gradient drives FO. The draw solution is treated to recover potable water and recycle the draw solute after absorbing leachate water (Wang et al. 2021).

#### Combined processes

Leachate treatment processes are often combined to achieve more effective and efficient treatment. The combination of these processes depends on the characteristics of the leachate, the regulatory requirements, the treatment plant's capacity, and the desired effluent quality. Some of the commonly used combined processes in leachate treatment.

Leachate transfer and biodegradation: One frequent and efficient approach to remediate leachate is the combination of transfer and biodegradation. Leachate is gathered from landfills through a variety of collecting systems and then transferred to treatment facilities. Pipelines or other transport mechanisms bring the collected leachate to a centralised treatment facility. By transferring ownership, the effects of leachate on the ecosystem can be mitigated. The leachate undergoes biological treatment procedures at the treatment plant. Microorganisms (bacteria and occasionally fungus) are used as the principal treatment technique to decompose organic chemicals in the leachate. Many other processes, such as the Activated Sludge Process (ASP), Sequencing Batch Reactors (SBRs), etc., can accomplish this biological treatment.

Chemical process and biodegradation: Treatment of leachate and mitigation of its environmental effects can be improved by integrating chemical and biodegradation procedures. The synergy between chemical processes and biodegradation is possible. By eliminating inhibiting chemicals or lightening the weight of non-biodegradable components, chemical procedures can assist in preparing the leachate for effective biodegradation. The leftover organic contaminants can be degraded further during the biodegradation process and turned into innocuous byproducts. Leachate may be effectively treated and its environmental impact reduced by adopting this integrated strategy.

Physical process and biodegradation: Combining physical processes with biodegradation is a successful strategy. To remove the solids from the leachate, physical methods such as screening, settling, and filtration are used. Microorganisms may decompose organic contaminants in both oxygen-rich and oxygen-depleted environments, respectively, to accomplish biodegradation. Using sequencing batch reactors or integrated fixed-film activated sludge systems to combine aerobic and anaerobic processes improves pollutant removal and lessens the environmental effect of leachate disposal. Biological treatment, such as “sequencing batch reactors” (SBR) or “integrated fixed-film activated sludge” (IFAS) systems, can be applied to the pretreated leachate once the physical processes are complete. The leachate in SBR or IFAS systems goes through cycles of aerobic and anaerobic conditions, which facilitates the effective biodegradation of a wide range of organic and inorganic contaminants. Biodegradable chemicals are helped along in the aerobic stage, while stubborn ones are eliminated in the anaerobic stage, which also results in the production of biogas (methane). The biological treatment process results in sludge, which can be stabilised and treated to lessen its negative effects on the environment.

#### Physical processes and chemical processes

The wide variety of contaminants in leachate may be effectively addressed using a holistic strategy that combines physical and chemical processes in leachate treatment. The specific process combination will be determined by the nature of the leachate and the quality criteria for the discharged or reused water. In order to get rid of larger particles and settleable solids, a typical treatment train may first use screening and sedimentation and then use chemical coagulation/flocculation to get rid of smaller suspended particles and colloids. After that, organic impurities and trace pollutants can be eliminated using chemical oxidation or activated carbon adsorption. The properties of the leachate and the legal requirements for the quality of the treated effluent will determine the precise mix of treatments. To get rid of solids and suspended particles, it uses a combination of screening, sedimentation, filtering, and air stripping. Contaminants can be removed by chemical processes such as precipitation, coagulation, chemical oxidation, ion exchange, and activated carbon adsorption. The environmental impact of leachate can be reduced by combining these techniques for efficient purification prior to safe disposal or reuse.

#### Combined leachate transfer, physico-chemical and biodegradation

An all-encompassing strategy for properly treating and minimising the environmental impact of leachate generated from landfills or waste disposal sites is the combined treatment of leachate, which involves a mix of leachate transfer, physico-chemical processes, and biodegradation. When water filters through garbage, it picks up a variety of pollutants and toxins; this mixture, called leachate, may be damaging to the environment if it isn’t adequately filtered out. Moving leachate from a landfill or other waste disposal site to a treatment facility is called leachate transfer. To avoid leachate from harming groundwater or surface water, it is common practice to direct it to a collecting system, such as a pipe or drain. Leachate may be efficiently treated, utilising a variety of treatment methods by transporting it to a central treatment facility.

To accomplish treatment goals and regulatory criteria, a mix of approaches is typically necessary, and this is determined by the characteristics of the leachate, the available budget, and the surrounding environment. Toxin removal performance ratings and an evaluation of treatment efficacy based on leachate age are displayed in Table 2, alongside the various landfill leachate treatment methods with merits and demerits, focus pollutants and average contaminants.

**Table 2:** Various leachate treatment methods

Treatment method	Techniques	Landfill leachate age (years)			Merit	Demerit	Focus pollutants	Average contaminants removal (%)	References
		Young < 5 years)	Medium (5–10 years)	>10 years					
Biological process	• Stabilization of pond/aerated lagoons	Good	Average	Poor	• Low cost to set up and maintain. •Ability to function in environments with varying organic contents •Effective removal of ammonia nitrogen	• The following are some limitations of biological treatment: It must be used with other treatment methods due to its poor efficiency in meeting regulatory requirements. • A large surface and temperature •sensitivity is required for maximum efficiency. •It stinks up the place. •Sensitiveness to heat or cold and pungent scents	Pathogens, phenolic chemicals, and both organic and inorganic substances	COD:40% H_4_-N:50	(Burman and Sinha 2020; Costa et al. 2019; Gao et al. 2015)
	• Activated sludge process	Average	Good	Poor	•Activated sludge treatment encompasses more than just lagoons. The best method in terms of efficiency and cost •Slightly stabilised sludge is produced, and the system may be adapted to communities of any size without compromising the safety of sensitive receiving regions. • Dephosphatation may be conducted rapidly and in tandem.	Problems include: • Excessive sludge production and inadequate settlement ability • The need for massive aeration • High capital costs and subsequent energy consumption • The requirement of regular monitoring and skilled personnel • Bacterial inhibition and sensitivity to hydraulic overflows; and • Frequent monitoring and skilled personnel.	Organic carbon, nutrients, and ammonia	COD:75%, NH_4_-N:70, BOD: 40%	(Gao et al. 2015; Gulsen and Turan 2004; Michalska et al. 2019; Yan et al. 2018)
	• Anaerobic digestion	Good	Average	Poor	•Low energy demand •Low surplus sludge production •Small reaction volumes with high purification yield •Biogas output and less phosphorus required	•Constraints on fluid movement due to refractory chemicals • Susceptibility to variations in pH and temperature •Noxious odours from digestate and ammonia •Heavy metals may cause digestive problems.	•Organic and inorganic matter	COD:85% NH_3_:82.92%	(Bove et al. 2015)
	• Microalgae biotechnology (phytoremediation)	Good	Average	Average	• The production of economically valuable biomass. • The use of ecologically friendly, low-cost carbon fixation. • The use of a sustainable nutrient and water source in the production of algal biofuels and bioproducts and • the use of a renewable energy source are all benefits of algal biofuels and bioproducts	•High needs for both water and power •Microalgae cannot survive in environments with high levels of ammonia (>500 mg/L).	Phenol, bisphenol A, 4-tert-butyl phenol, EDCs, PPCPs, antibiotic resistance genes, SS, perfluoroalkyl and polyfluoroalkyl substances, organic compounds, metals	N–NH_3_ :70%	(Chang et al. 2018, 2019; Li et al. 2019)
	Moving Bed Biofilm Reactor (MBBR)	Good	Average	Average	• Sludge dispersion is decreased, and it is easy to use and resistant to high amounts of ammoniacal nitrogen .• Low sensitivity to hazardous chemicals • Fast sludge settlement • Greater biomass concentrations in the reactor	• Expensive to start up and maintain	COD and ammonium	COD: 60–81%	(Luo et al. 2020; Renou et al. 2008)
	Biological filters (Trickling filters)	Average	Average	Poor	•Filter media is inexpensive and very effective in removing pollutants and •The system is easy to use.	• The filter’s top absorbs more N-NH3, stifling nitrifiers and preventing bacterial growth. • Clogging issues anytime there is a high concentration of organic matter	SS, COD, BOD, NH4 +-N, and turbidity	COD: 44% BOD: 60%, N–NH_3_: 15% Turbidity: 30%, SS:70%	(Renou et al. 2008)
	Upflow anaerobic sludge blanket (UASB)	Good	Average	Poor	• High efficiency in treating organic waste and converting it	Low hydraulic retention time • Vulnerability to poisonous substances	-	-	(Gao et al. 2015; Kurniawan et al. 2006; Palanisamy et al. 2019)
Physical methods	Fungal treatment	Good	Average	Average	•Positive effects on leachate treatment during landfill lifetime		Acids, lignin, cellulose and hemicellulose		Luo et al. (2020)
	Coagulation-flocculation	Good	Average	Average	•Consolidated idea that is both simple and inexpensive. •Flexible in its operations; best used as a biological pre-treatment. •Distinguishing features include these. •Inexpensive and effective waste-water treatment	•Coagulants are expensive. •There is a limit to how much COD can be removed. •Sludge production is high, creating secondary pollution. •Aluminium and iron concentrations may rise.	Non-biodegradable organic matter, clays, colloids, suspended solids, surfactants, heavy metals and acids	COD:10–50% DOC:93% Colour:83%	(Aziz et al. 2018; Kamaruddin et al. 2017; Miao et al. 2019; Trabelsi et al. 2013)
	Precipitation	Poor	Average	Poor	• Feasible to reuse waste materials as fertilisers. • To conclude, the costs are lower than for other physical-chemical processes. • The time required is less than for biological processes, and the entire process is more efficient.	• It is very difficult to locate precipitating agents, and the effectiveness of the method is limited by the pH ranges employed in its application.		NH_3_:90% organic matter: inefficient.	(Tugtas et al. 2013)
	Adsorption	Good	Average	Poor	• A method that is both effective and encouraging. Surface reactivity, surface area, microporous structure, adsorption capacity, and thermal stability are all enhanced.	• Granular/powdered activated carbon is expensive. • It must be regenerated at regular intervals • It cannot be used alone to treat leachate. • Carbon fouling is a possibility.	Organic and inorganic pollutants, recalcitrant organic compounds, heavy metals	COD:50% colour:88 chromium:99%	(Bu et al. 2010; Kamaruddin et al. 2017; Saxena et al. 2019)
	Air stripping ➢ Ammonia stripping ➢ Methane stripping	Poor	Average	Average	• Increasing a process's pH, temperature, and retention time can substantially impact efficiency. • Ammonium stripping is cost-effective.	• The generation and release of contaminated gases (NH3). • The need for additional ammonia control for exhaust air. • The necessity of using a large stripping tower to solve foaming problems. The necessity of scaling the stripping tower with calcium carbonate;	Methane, ammonium NH3–N, and volatile organic compounds (VOCs)	NH_3_-N:99.5 % COD: poor	(Dogaris et al. 2020; Kurniawan et al. 2006; Saxena et al. 2019)
	Membrane filtration	Good	Good	Good	• Functionality • High rejection of oxidising agents and organic solvents. Advantages of reverse osmosis include increased flow and less energy use. • The pressure needed for this method is lower than reverse osmosis.	• The average lifespan of a membrane is just about 5 years. •It’s important to keep up with routine maintenance. •Costs are high at the outset.	Suspended solids and colloids	OD: 89% BOD: 92% N–NH_3_:97%	(Dabaghian et al. 2018)
	Ultra-filtration (UF)	Good	Average	Good	• Can eradicate bulk molecular weight compounds that appear to clog the membrane of reverse osmosis • High efficiency with low operating costs	• Incomplete removal of polluting substances • Reduced applicability due to fouling of the membrane	High molecular weight compounds		(Abuabdou et al. 2020; Renou et al. 2008)
	Nano-filtration (NF)	Good	Average	Poor		• Pricey	Organic and inorganic matter, heavy metals, recalcitrant organic		(Dabaghian et al. 2018; Renou et al. 2008)
	Reverse osmosis (RO)	Good	Average	Average	• It is functional throughout a wide temperature and pH range and produces high fluxes	• Not economically appealing • Extensive pretreatment is required before RO • Membrane fouling • High-energy consumption • Generation of large volume of concentrate	Organic and inorganic dissolved compounds, heavy metals, suspended and dissolved solids	Organic contaminants: 99.6%. Flux recovery> 80%	(Kurniawan et al. 2006; Luo et al. 2020; Renou et al. 2008)
	Microbial fuel cell	Good	Good	Good	• Eco-friendly (low emission of toxic gases) Recovery of valuable compounds from the substrate. •Alternative source of fuel	• If discarded, the microbes and toxic chemicals found in leachate can cause harm to the environment. • Low power output		NH_3_-N: 89.7% COD: 98.47% Nitrite: 99% Phosphate: 70%	(Qi et al. 2019; Wang et al. 2021)
Chemicals	Chemical precipitation	Good	Good	Average	• Low initial investment due to the use of cheaper equipment and a straightforward procedure	•Low COD removal efficiency necessitates a lot of chemicals and a lot of precipitants. • The process is sensitive to pH changes and produces a lot of sludge that needs to be disposed of. • •Efficiency controlled by the molar ratio of ${\textrm{PO}}_4^{3-}$, Mg and ${\textrm{NH}}_4^{+}$	NH_3_–N, ${\textrm{NH}}_4^{+}$-N, heavy metals, and non-biodegradable organic compounds		(Choudhury et al. 2021; Kamaruddin et al. 2017; Renou et al. 2008)
	Chemical oxidation	Average	Good	Poor	• Leachate contains organic compounds that are oxidised to their most oxidised form.	•High oxidant dosages, investment cost, electrical energy, and • The production of surplus sludge is necessary • It is doubtful that the wide variety of contaminants present will be effectively dealt with.	Non-biodegradable, soluble organic, and toxic substances		(Dogaris et al. 2020; Gao et al. 2015; Luo et al. 2020)
	Advanced oxidation processes (AOPs)	Good	Average	Average	• Effective approach for stubborn organic mineralization in leachate • One-pot technology that functions at room temperature and pressure	• The chlorine oxidation potentials typically reduce treatability. • Large-scale effluents cannot afford the high oxidant dosages, capital expenses • Electricity is needed for these systems. • They also produce an excessive amount of sludge.	Non-biodegradable and toxic organic compounds		(Foo and Hameed 2009; Gao et al. 2015; Chuangcham et al. 2008; Renou et al. 2008)
	Fenton process	Good	Poor	Average	•Successfully utilised to mineralize a wide range of organic components in leachate •Demonstrates considerably quicker kinetics than biological treatment	• Embroidered by the final iron sludge output requiring ultimate disposal • Safety and operational hazards associated with high acid requirements • Incures high treatment cost	Organic constituents		(Luo et al. 2020)
	Photo-Fenton	Poor	Poor	Average	• Involves the depletion of Fe^3+^ to Fe^2+^ coupled with ferric carboxylates photo-decarboxylation	• Results in very significant treatment costs			(Luo et al. 2020; Umar et al. 2010)
	Electrochemical oxidation				•Mineralizes organic substances into CO_2_ and water • Effective for disintegrating nonbiodegradable contaminants •Provides high efficiency with no sludge production; increases biodegradability index (BOD/COD). •operates easily in various settings; is amenable to automation •has a low environmental impact.	• Consumption of a lot of energy • High running expenses • The possibility of the creation of chlorinated organic compounds	Colour, organic contaminants, BOD and COD, ammonia nitrogen		(Gao et al. 2015; Mandal et al. 2017)
	Electro-Fenton processes				• Suitable for the treatment of leachates containing exceptionally high concentrations of organic load	• Using electricity and ultraviolet light increases energy consumption and infrastructure expenditures.	Organic matter: ammonia nitrogen		(Luo et al. 2020; Yaqoob et al. 2021)
	Electro-coagulation				•Cost-effective operation and upkeep •Improves the flocculation process so that less sludge with higher •Hydrophobic solid content may be generated without using chemicals.	Energy-intensive • Passivation of electrodes • Formation of hazardous chlorinated by-products and an impenetrable oxide layer	COD, TSS, phosphorus		(Fernandes et al. 2015; Propp et al. 2021)
Leachate transfer	Co-treatment with sewage				• A workable, practical, hassle-free, and inexpensive option • Boosts the Biological Oxygen Demand/Carbon Oxygen Demand ratio, making the wastewater biodegradable. • Leachate, sewage nitrogen, and phosphate work well in treatment.	• Some resistant organic molecules (humic acids, fulvic acids, and hydrophilic) in leachate can escape. • Heavy metals and refractory chemicals suppress the breakdown of microorganisms in the activated sludge process. • Reduce the waste streams’ UV transmittance and hence hinder the effectiveness of disinfection. Sludge generation in urban wastewater treatment plants is raised due to increased organic leachate load and a lack of alkalinity.	BOD, COD, ${\textrm{NH}}_4^{+}$-N, suspended solids		(Dogaris et al. 2020; Ganguli et al. 2016; Luo et al. 2020; Trabelsi et al. 2013)
	Irrigation by spraying				•Efficient methods for refining high-volume, low-strength leachate and treated leach	•Large areas of vegetation around dump sites are inaccessible. • Volatile pollutants and aerosols are created, and leaf and plant attrition is threatened. • The capacity to decrease organics is limited.			(Schiopu and Gavrilescu 2010)
	Recycling				• Improves leachate standard • Shortens stabilisation time • Reduces leachate volume • Is easily operated, pH-buffering, and cheap • Increases moisture content above their field capacity and provides nutrient and enzyme transfer between the methanogens and the liquids/solids	• It has the potential to impede methanogenesis. • Solid waste decomposition in anaerobic settings may be impacted by recirculated leachate volumes that cause ponding, saturation, and acidity. It serves no use and has no commercial appeal.	COD, BOD		(Gao et al. 2015; Kamaruddin et al. 2017; Renou et al. 2008; Schiopu and Gavrilescu 2010)
	Evaporation				•Reduces the volume of leachate to a concentrated residual volume that is easy to dispose of while maintaining quality.	•Facing smell, gas aggregation, process operation, and maintenance issues			(Schiopu and Gavrilescu 2010)

##### Bioelectrochemical systems

**Fig. 4:**
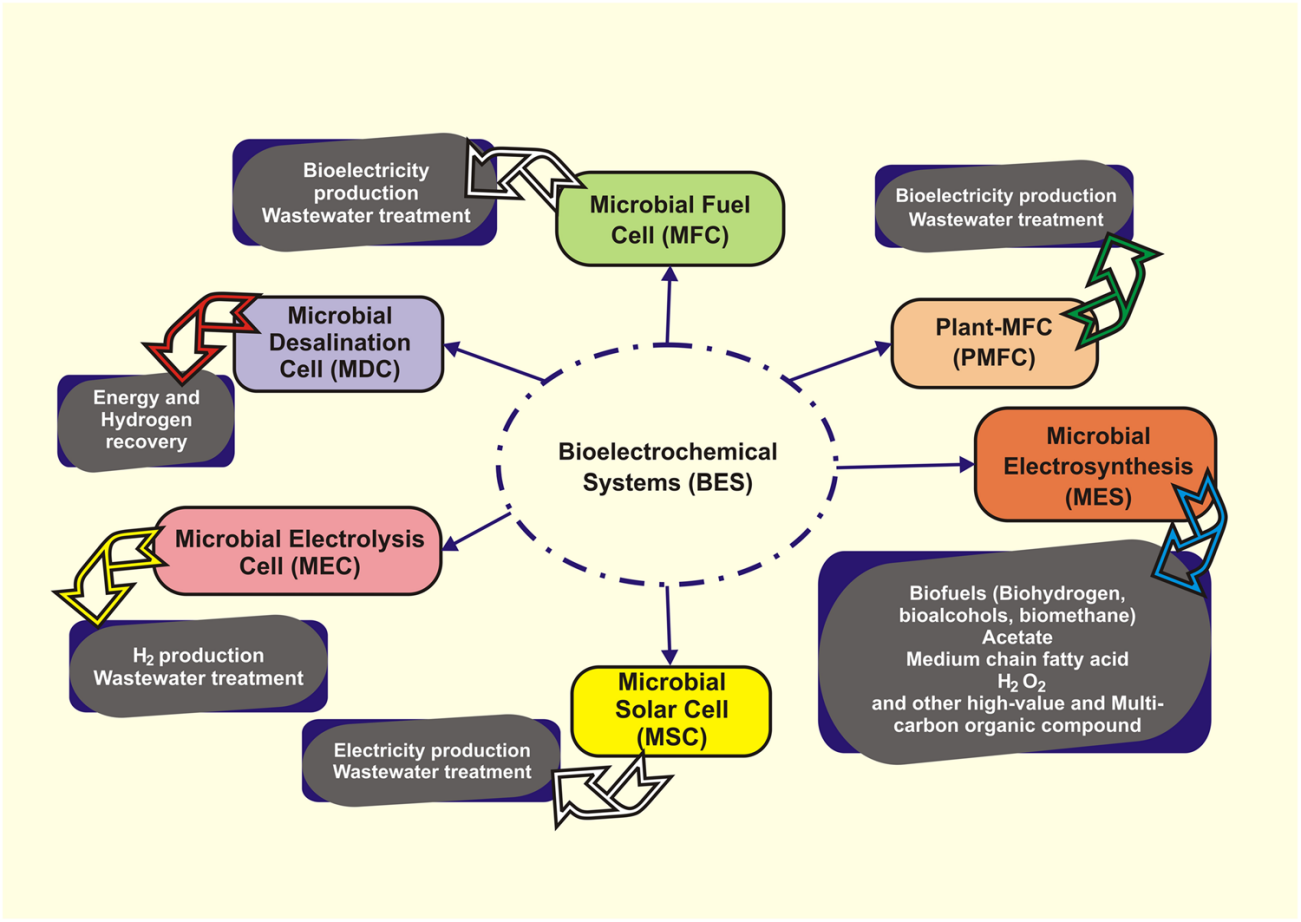
Overview of BES categories by application mode (Quraishi et al. 2021)

The chemical energy held in biodegradable materials is converted into electric current and chemicals by microorganisms in a bioelectrochemical system (BES). BES offers a new way to manage waste while recovering energy and materials (Logan and Rabaey 2012; Zhang et al. 2020) due to its adaptability as a platform for oxidation and reduction reaction-oriented operations. There are a variety of designs of BES reactors available for various uses, but typically, they consist of an anode, a cathode, and a separator (though the separator is optional). Microorganisms oxidise organic matter, such as wastewater, in the anode chamber of a microbial fuel cell (MFC), generating electron flow (current) to the cathode, where the electrons can be used for direct electricity production or the reduction of water or oxidised chemicals (in a microbial electrolysis cell (MEC) or microbial electrosynthesis (MES). Fig. 4 describes the classification and applications of BES.

BES technologies, including MFCs, MECs, and MDCs, are frequently used for landfill leachate treatment (Zhang et al. 2019). The MFC has an anode and cathode compartments separated by an ion-selective membrane (Arends et al. 2012; Logan and Rabaey 2012; Schroder 2011; Mook et al. 2013; Schroder et al. 2015; Elmaadawy et al. 2020a).

**Fig. 5:**
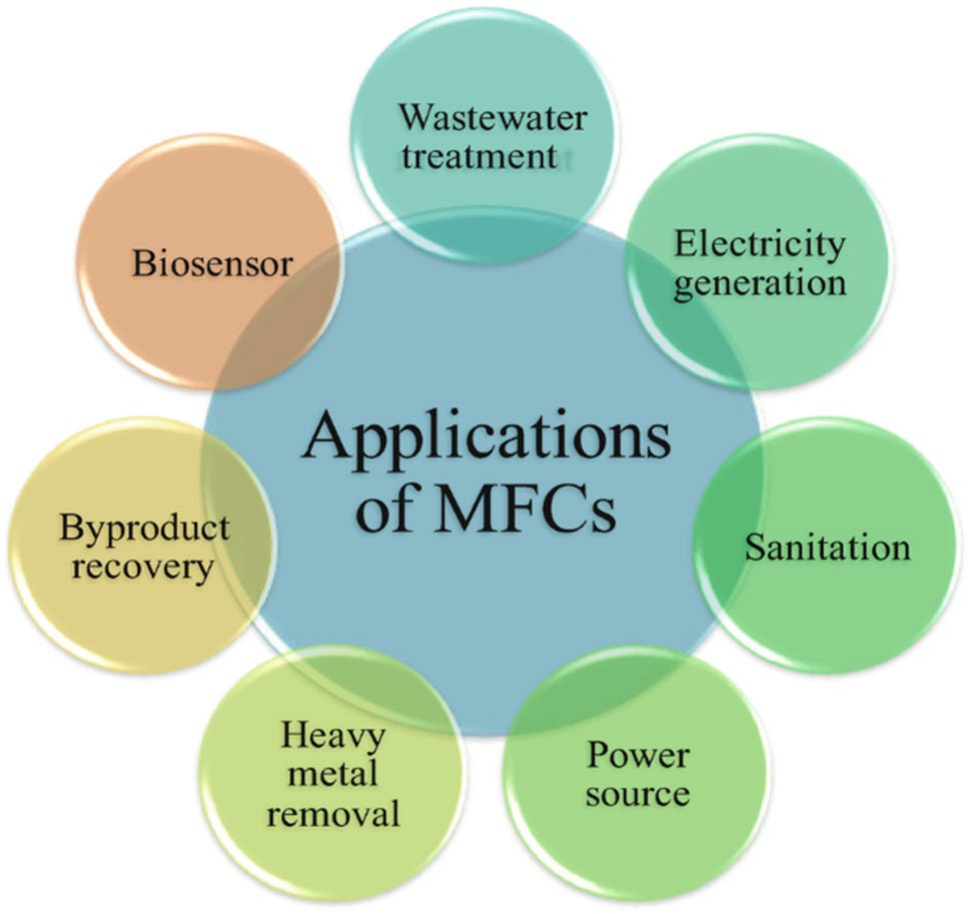
Functions and advantages of microbial fuel cell

MFC degrades organic substrates (electron donors) by anaerobic oxidation reactions by various microorganisms at the anode compartment. Electroactive bacteria or mediators deliver electrons and protons to the cathode compartment (Sun et al. 2016; Scott and Yu 2015; Logan 2008). At the cathode, oxygen is reduced to water, generating bioelectricity from electrons and protons. Different redox potentials between the cathode and anode drive the process (Scott and Yu 2015; Logan 2008). MFC produces clean energy and produces 2.4–26.5 times less sludge than aerobic-activated sludge (Cheng et al. 2011). Fig. 5 highlights the predominant advantages of MFC technology.

Leachate from landfills can be used as a fuel in MFCs for energy recovery due to its high organic matter content and the presence of various inorganic metals in its composition, both of which contribute to its high electrical conductivity and thus increase power generation (Damiano et al. 2014; Iskander et al. 2016). Tao et al. (2015); Virdis et al. (2010); Yan et al. (2012); Li et al. (2016); Almatouq and Babatunde (2018); Liu et al. (2017); Zhang et al. (2019); Vijay et al. (2019) are just a few of the recent publications that have focused on the use of microbial fuel cells to remove nutrients from municipal wastewater. Due to its high ammonia nitrogen content, landfill leachate may reduce the efficacy of biological treatments. Only a few studies have reported ammonia removals by standalone MFCs (Damiano et al. 2014; Huang et al. 2018; Hassan et al. 2018), yet their low treatment performances and low output power densities have triggered the investigation of hybrid treatment process with higher treatment efficiency (simultaneously carbon and nitrogen removals) and bioelectricity generation (Nguyen et al. 2017; Hassan et al. 2017; Elmaadawy et al. 2020a).

In studies of real-world leachate treatment, anaerobic treatment or repeated treatment techniques were shown to be effective in the literature. Additionally, it is well known that the anaerobic state of the MFC anode has shown effective in treating actual landfill leachate (Zhang et al. 2015; Hassan et al. 2018). However, the high strength of organic and ammonium content in Landfill leachate that might hinder the microbial activity of the cathode limits the infusion of undiluted leachate into the cathode chamber. Some research has even recycled the effluent from treating leachate in the anode back to the cathode for further treatment and ammonia reductions (Nguyen et al. 2020). For this reason, the viability of leachate treatment in both the anode and cathode is essential for actual field applications and sustainability, which may be encouraged by adopting MFC technology (Elmaadawy et al. 2020a).

##### Organic and nutrient removal mechanisms

In all microbial systems, nutrients are essential for cell growth and proliferation. When individuals say “nutrient removal,” they imply removing nitrogen and phosphorus from wastewater or landfill leachates (Kang et al. 2008). High nitrogen and phosphorus effluents harm the environment and increase river eutrophication. Microbial fuel cells and other biological, chemical, and bio-electrochemical therapies can eliminate them. Understanding their removal processes and identifying system performance factors is essential for improving and selecting the best microbial fuel cell solution for their removals. Variations in landfill leachate characteristics and pollutant concentrations may affect nutrient removal and system effectiveness. Inorganic anions, including carbonate, sulfate, and chloride, leachate age, dissolved organic matter (DOM), and ultraviolet quenching material might alter contaminant removal procedures. MFC removes organics mostly by anaerobic breakdown in the anode compartment. Leachate contains a lot of ammonia, like nitrates, which can be removed by microbial activity or abiotic reduction at the cathode electrode. Leachate composition and operation impact effluent organic compounds. Hassan et al. (2018) found that batch leachate with a 60% dilution ratio removed more than continuous and undiluted leachate with the same conditions. The presence of chloride anions in leachate helps remove organic debris and ammonia nitrogen and creates active chlorine in the electrochemical oxidation process (Turro et al. 2012; Pérez-Pérez et al. 2012). Iskander et al. (2017) examined UV quencher removal and hydraulic retention time, anolyte recirculation rate, and external resistance. The combination of MFC with granular activated carbon adsorption reduced UVQS by 89.7% and organic reduction by 75.3%. MFC plants remove leachate ammonia via direct microbial oxidation or cathode reduction. NO3 concentration and aeration frequency affect removal efficiency and efficacy (Zhang et al. 2016; Ye et al. 2016). The following section discusses microbial catalysts at the anode and cathode electrodes to remove carbon and nutrients.

##### Microbial fuel cell treatment technologies

The utilization of Microbial Fuel Cells (MFC) for the treatment of landfill leachate is regarded as a novel and environmentally friendly technology. This approach offers the additional benefits of generating bioelectricity and producing high-quality effluent (Sun et al. 2016; Hassan et al. 2018; Feng et al. 2015; ElMekawy et al. 2015). Various configurations of microbial fuel cells (MFCs) have been investigated for their efficacy in optimizing power generation and organic removal in landfill leachate. These configurations include single-chamber MFCs, double-chamber MFCs, upflow MFCs, and stacked MFCs. The study focused on the investigation of the different types of MFC systems with varying designs, operational variables and modes of operations to assess the efficacy of the system for treatment and power generation.

##### Design of MFCs

The success of a microbial fuel cell (MFC) relies on the customization of various factors such as reactor volume, oxygen supply, membrane area, and electrode spacing. These parameters play a significant role in controlling the performance of MFCs. The reactor’s design, including its size, shape, and configuration, is a crucial aspect that can greatly impact the efficiency and overall success of the MFC. Different designers can choose and modify these design elements according to their specific requirements, resulting in variations in MFC designs. The careful consideration and optimization of the fuel cell design are essential for achieving desirable power generation and microbial fuel cell performance outcomes. In addition, the design of MFCs can vary based on specific applications, scalability requirements, and desired power output, and ongoing research focuses on developing novel materials, improving electrode architecture, and optimizing the system configuration to enhance the efficiency and practicality of MFC technology (Flimban et al. 2019).

There are three types of MFCs: Single chamber MFC, double chamber MFC, and stacked MFC. However, only single and double-chamber MFCs were considered for this study, as seen in Fig. 5A–B.

##### Single chamber MFC

A single chamber MFC’s anode and cathode electrodes (Fig. 5B) are typically positioned in the same air-cathode exposed compartment. A single-chamber reactor may be the more difficult to construct of the two options. The basic MFC prototype can contain one or two chambers depending on how the anode and cathode are built. The MFC prototype has undergone structural and design changes in addition to these two main layouts. Most research into Single-chamber MFC has focused on its use in producing energy and eliminating organic pollutants. The generated energy had a volumetric power density of 10,000 to 20,000 mW/m^3^, and the coulombic efficiency ranged from 1 to 80%. This view is supported by evidence from numerous sources (Barelli et al. 2018; Hernández-Flores et al. 2017; Vázquez-Larios et al. 2015). The influent substrate type significantly impacts MFC bioreactor performance and bioelectricity generation. Hernández-Flores et al. (2017) evaluated the air cathode single chamber MFC (SCMFC) supplied with municipal wastewater, and in phase 2, a combination of municipal wastewater and landfill leachate (MWW/LFL) for bioelectricity generation and organic removal. Increased CE of 47.5% and COD removal efficiency of 80% were seen with mono-substrate MWW, while the highest COD removal of 86% was achieved with the MWW/LFL combination. MFC with a high organic substrate of single leachate substrate had the highest power density of 489 mW/m^3^, surpassing single substrate MWW (315 mW/m^3^) and MWW/LFL combination (82 mW/m^3^). Hernández-Flores et al. (2017) compared power density and organic removals of SCMFCs fed with three different LFL/SR-I ratios (Mixture1: 30%LFL/70% SR-I, Mixture2: 70% LFL/30% SR-I, and Mixture 3: 50% LFL/50% SR-I) and two exchange membranes (zirfon and Nafion). SCMFC with 50% LFL/50% SR-I Zirfon membrane showed better power density (10,380 mW/m^3^) than Mixtures 2 (8050 mW/m^3^) and 3 (4260 mW/m^3^). In Mixture 1, SCMFCs with NF membranes removed more COD than Mixtures 2 and 3, with average values of 68.42%, 64%, and 48.11%, respectively. The results demonstrate the significance of choosing the right membrane and substrate compositions for optimal power output Elmaadawy et al. (2020a)

##### Double chamber MFC

Anode and cathode chambers, typically separated by an ion exchange membrane, are standard components of dual-chamber MFCs (Fig. 5A). Concurrently blocking the entry of oxygen into the anode. As a result, this setup is frequently employed to treat wastewater and produce power. A proton exchange membrane (PEM) serves as the principal proton transfer medium between the anode and the cathode, completing the circuit between the two chambers (Fig. 3A). This seals off the cathode from any more oxidizers or oxygen and concludes the reaction. Removal efficiency for double chamber MFCs was between 40 and 90%, with power densities of 87–158 mW/m^2^. The double chamber’s power boost is due to the membrane’s facilitation of electron transport from the anode to the cathode (Yan et al. 2018). Özkaya et al. (2013) investigated the effect of OLR (0–200 gCOD/L. day) on power density. The power density increased gradually with increasing OLR (0–67 gCOD/L. day), peaking at 2250 mW/m^3^ (900 mW/m^2^) at 67 gCOD/L. day. As loading rates increased up to 200 gCOD/L. day, power density decreased due to the high biodegradability of leachate and the continuous flow mode of operation (Elmaadawy et al. 2020a). The COD removal efficiency improved to 35–40% when the influent COD concentration rose from 1000 to 50,000 mg/L. Consistent with Greenman et al. (2009) and Elmaadawy et al. (2020), OLR ranged from 0.3 to 2.9 kg BOD5 m^−3^ day^−1^, with the highest power density (0.26 mW/m^2^) at 0.8 kg BOD5 m^−3^ day^−1^. Moharir and Tembhurkar (2018); Elmaadawy et al. (2020a, b)

Found that increasing the influent COD concentration from 500 to 1250 mg/L improved the output power density of leachate. The maximum power density was 29.23 mW/m^2^ at 1250 mg/L COD due to higher biodegradability and a significant pH difference between the anode and cathode chambers, which increased proton movement through the membrane. Conversely, COD removal was highest (72.2%) at 500 mg/L influent COD concentration and during anolyte recirculation, which increased microbial activity and organic removal. MFC architecture design can improve system performance and power density by reducing internal resistance through electrode distance, recirculation, and number. You et al. (2006) conducted a comparison of SCMFC and DCMFC for treating LFL. The DCMFC showed a minor increase in columbic efficiency (CE) without improving power densities, likely due to higher membrane separator internal resistance.In contrast, SCMFC had lower CE despite a 3.4-fold higher output power density than DCMFC. The right anodic and cathodic pH affects MFC performance and power output. Li et al. (2019) examined how anolyte ionic strength and pH affect MFC output energy and COD removal, using carbon felt for anode and cathode and feeding synthetic food waste leachate in batch mode. Results indicate that the highest power density (1000 mW/m^3^) was attained at 0.1 mol/L NaCl with 371Ω internal resistance, whereas the highest COD removal (85.4%) was seen at 0.15 mol/L NaCl. The progressive increase in anodic pH from 4 to 9 led to a maximum power density of 9956 mW/m^3^, decreased internal resistance of 35.3 Ω, and 80% COD elimination efficiency.

**Fig. 6:**
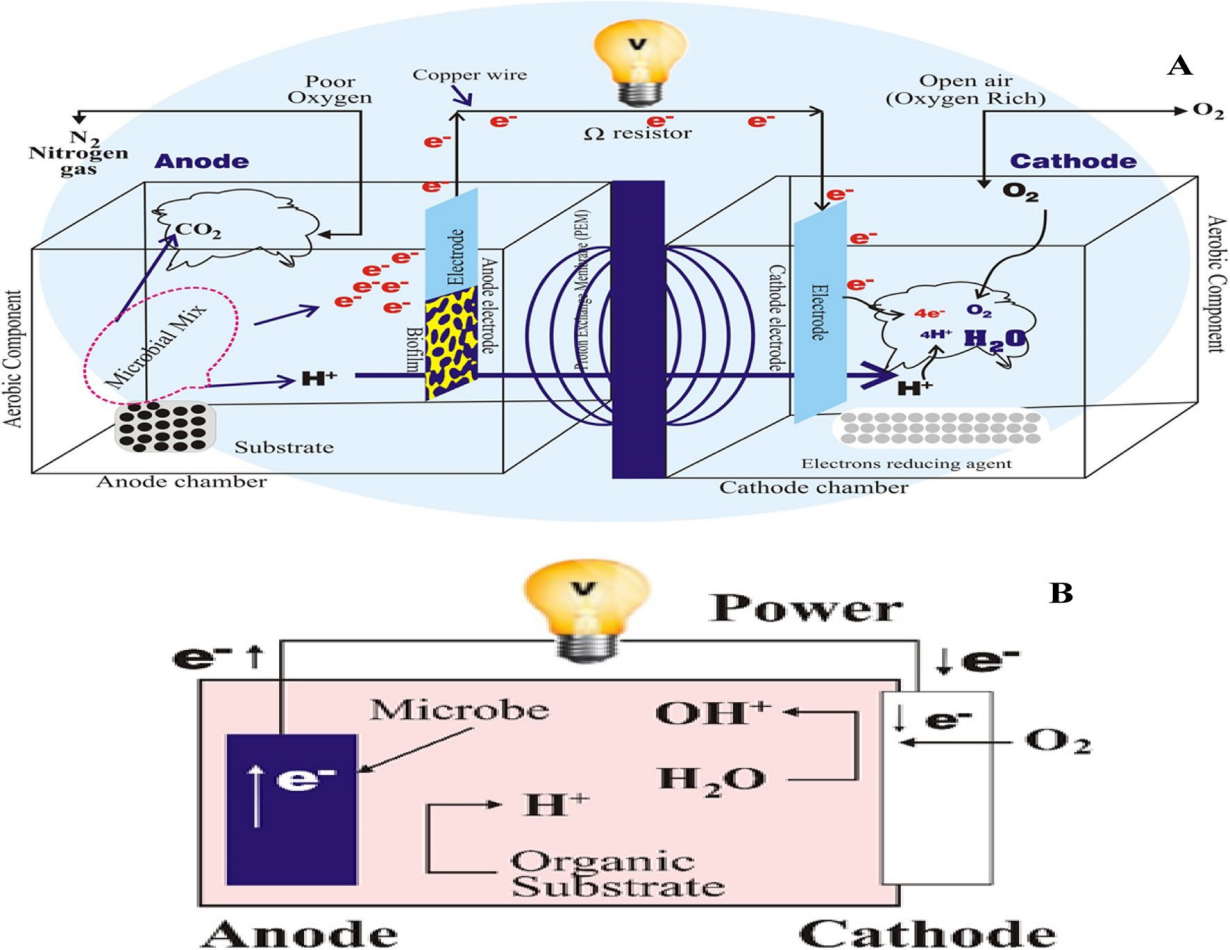
(**A**) and (**B**) represent the Double chamber and single chamber MFC schematic diagrams, respectively

Furthermore, in a single-chamber MFC, the anode and cathode are placed within the same chamber, resulting in a simpler design and reduced complexity. This design allows easy operation, maintenance, and direct access to the electrodes for monitoring and sampling. Single-chamber MFCs also have a higher power density and are more suitable for low-strength wastewater treatment. However, a disadvantage of single-chamber MFCs is the possibility of electrode fouling or cross-contamination between the anode and cathode compartments, limiting their long-term stability and efficiency. On the other hand, double-chamber MFCs separate the anode and cathode into distinct chambers, mitigating the issues of cross-contamination and electrode fouling. This design allows for better control over the electrochemical reactions and offers higher coulombic efficiency. Double-chamber MFCs are suitable for higher-strength wastewater treatment and have the potential for improved long-term stability. However, the double-chamber configuration introduces additional complexity, requiring ion-selective membranes for proton transport and necessitating a more sophisticated setup. Additionally, double-chamber MFCs typically have lower power densities compared to single-chamber MFCs. Therefore, the choice between single-chamber and double-chamber MFCs depends on the specific application, desired performance, and trade-offs between simplicity, power density, and long-term stability Fig. 6.

Anodic reactions:

1 $${\textrm{C}}_{12}{\textrm{H}}_{22}{\textrm{O}}_{11}+13{\textrm{H}}_2\textrm{O}\to 12\textrm{C}{\textrm{O}}_2+48\textrm{H}++48\textrm{e}-$$

Cathodic reaction:

2 $${\textrm{O}}_2+4\textrm{e}-+4\textrm{H}+\to 2{\textrm{H}}_2\textrm{O}$$

## Fundamental of stacked chamber MFC

A stacked microbial fuel cell configuration is a highly inefficient way of increasing power output. Numerous factors, including cell number, connection type, variable loads, and electrolyte flow rates, impact the performance of a stacked MFC (Zhang et al. 2017). One study found that a stacked MFC featuring a serpentine flow field achieved the highest maximal power in a series connection, surpassing the power in parallel and hybrid connections (Arwa et al. 2016). In another study, researchers created a paper-based MFC stack that could be integrated and stacked by folding filter paper and connecting multiple MFCs in series. This paper-based MFC stack generated a power density two orders of magnitude higher than previous reports. A stacked MFC was also evaluated for its viability in a septic tank and demonstrated promising results in terms of both electricity generation and chemical oxygen demand removal.

**Fig. 7:**
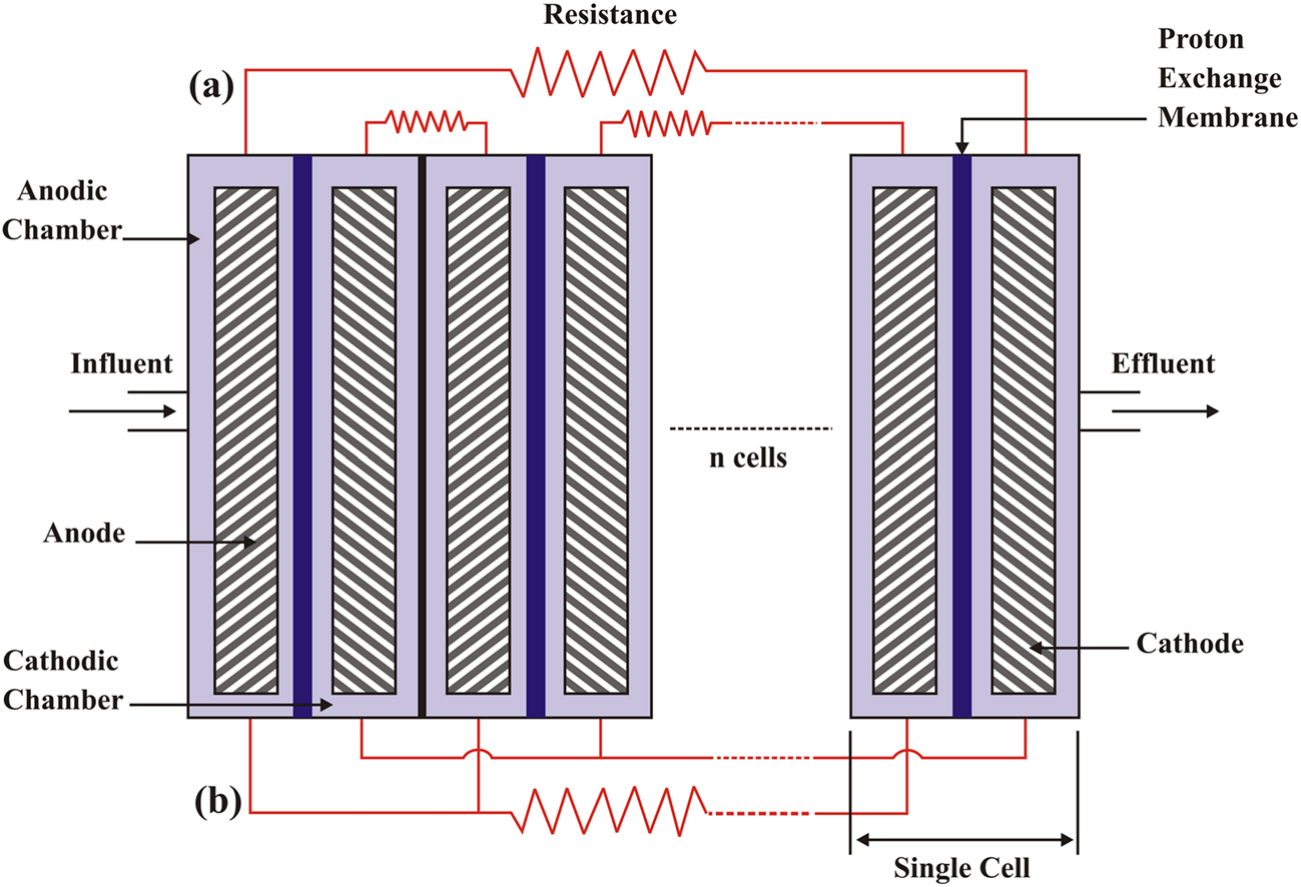
Schematic diagram of stacked MFC

Moreover, a stacked passive direct-methanol fuel cell (DMFC) set designed with polymer bipolar end plates was suggested, featuring a novel structure and higher mechanical strength (Liliana et al. 2016). Finally, a stacked solid-oxide fuel cell (SOFC) was developed, integrating seal parts to ensure mechanical strength and efficient fuel and air gas circulation. The illustration of stacked MFC is shown in Fig. 7

## Documented studies on the MFCs performance using leachate as a substrate

The results of previous trials, ranging from quick lab tests to in-depth pilot projects, were compiled using a systematic, deliberate approach. This part evaluates the current knowledge on applying landfill leachate to MFCs and looks ahead to potential developments in the field. Table 3 shows that operating modes, reactor types, catalyst, inoculum, internal resistance, microbial activity, electrodes, membranes, operational parameters, design, and configurations do not affect the effectiveness of MFCs as a leachate treatment and power production tool. High levels of organics removal, including NH_4_-N, COD, and BOD_5_, are shown in Table 2 for MFCs with twin chambers and continuous batch working mode. The diffusion of protons is aided by an ion exchange membrane that prevents the exchange of solutions and oxygen between the two compartments (Lee et al. 2013). Consistent with the findings of the research above (Sami et al. 2019), Table 3 shows that the power potential is greatest in a single chamber due to the shorter distance between the electrodes. Double-chamber MFCs may not perform as well as single-chamber MFCs because of the higher distance between electrodes. However, the type of influent substrate is a crucial part of MFC bioreactors since it affects MFC performance and bioelectricity production. The power density was suppressed at high substrate concentrations (Liu et al. 2015). Substrate composition is determined by the concentration of refractory organics (NH_4_-N); a high concentration reduces the efficiency of MFCs. Ammonia nitrogen, abundant in landfill leachate, has inhibited microbial activity in biological treatment (Ali et al. 2020). Ammonia can be eliminated using freestanding MFCs; however, just a few studies have proven this. The microbiological process of electron transfer from the anode to the cathode chamber is slowed by leachate from landfills with high concentrations of ammonium compounds, as shown in Table 2. Temperature changes also directly affect this; refractory organics are eliminated much more during the thermophilic stage than during the mesophilic stage (Table 3). Researchers have found that MFCs’ ability to remove COD and generate power is significantly impacted by temperature. There was only a small drop in power density (9%) when the temperature was lowered from 32 to 20°C (Lu et al. 2009). Temperature impacts vary depending on the substrate, and temperature changes are substantial for complex substrates like landf Ahn and Logan (2010), covered by Ahn and Logan (2010), who studied MFC performance at ambient (23°C) and mesophilic (30°C) temperatures. Recently, the results of batch tests on single and double-chamber MFCs at temperatures ranging from 4 to 35°C were reported (Larrosa-Guerrero et al. 2010). With an efficiency of 84% in removing COD and a power density normalized to the anode surface area of 34.38 mW/m^2^, the MFC performed best at a temperature of 40 °C. For both dual and single chambers, most previously observed temperatures fall within the mesophilic (23–40°C) range (see Table 3). Internal resistance drops with increasing temperature (Behera et al. 2011). The duration of the hydraulic retention time (HRT) is critical in the generation of electrical power. As the time interval lengthens, so does the power potential. As can be observed from Table 2, HRT has a negative impact on MFC performance in any reactor mode. Hydraulic retention time (HRTs) affect biological therapy, leading to power generation shifts during treatment. The removal efficiency of COD and T-N was shown to decrease, and electricity generation decreased when HRTs were shortened (Chang and Herrmann 2018). COD elimination effectiveness dropped from 96.28% at an 8-h HRT to 90.67% at a 5-h HRT, as reported by (Chang and Herrmann 2018). The efficiency with which total nitrogen was removed decreased from 74.16% during an 8-h HRT to 53.42% during a 5-h. The system suffered when HRT was reduced to 0.25 day (OLR 200 g COD/L/day), as COD removal efficiency fell below 5%. The voltage and current in the cells also dropped to negligible levels. A few pilot-scale MFC systems and a rising number of 1 to several hundred litre-scale up studies have come from recent advancements in materials and reactor designs. The transition of this method from the lab to the pilot scale is a major step toward its eventual commercialization. Single-chamber MFCs, double-chamber MFCs, up-flow MFCs, and stacked MFCs are only some MFC designs investigated for usage in landfill leachate for power generation and organic removal. Most studies on landfill leachate treatment concentrated on power production and organics removal despite the reduced cost and simplicity of single-chamber MFCs, and only a few researchers measured ammonia and nitrogen removal. Electrode material and surface area, inoculation type, and separating membrane type all influence power densities in MFCs used to treat landfill leachate (Table 3). High organic leachate substrate, influents of COD:12,300 mgNL1 and TAN: 2900 mgNL1, and platinum (pt) catalyst resulted in the greatest power density in a single chamber of 1799 mWm^3^ (Table 3). The MFC’s extraordinary performance was attributed to the Pt catalyst. The results corroborate those of (Hernández-Flores et al. 2017), who found that energy may be generated from a single chamber fed with fresh leachate. Electrodes play a dual role in the system, acting as a carrier surface for the microbial population and as an electron donor or acceptor, depending on the kinetics of pollutant removal. Hence, their surface area substantially impacts system performance and bioelectricity production. Energy output and system performance benefit from increased electrode surface area (Sonawane et al. 2017). Activated carbon, zinc electrode, and black carbon have varied power densities depending on their surface area, as shown in Table 3 (Alabiad et al. 2017). Activated carbon has the potential to remove ammonia at a 96.6% efficiency. When it comes to utilization in power plants, though, zinc shines. This conclusion was consistent with an earlier one by Gálvez et al. (2009). Rectangular, cylinder upflow, cylinder U, H type and circular MFCs with two chambers have all been designed and applied to treat landfill leachate (Du et al. 2007). Many studies have compared single and dual chambers, but it isn’t easy to conclude them because each experiment has a different operating strategy and uses various measuring units. When comparing a Single chamber MFC to a Dual-chamber MFC for treating landfill leachate, You et al. (2006) discovered that the Dual-chambered supported a slight rise in columbic efficiency (CE) without increasing energy densities. This supports the results of the literature review shown in Table 2. An environment’s pH significantly affects bacterial activity, decreasing efficiency and power output. Most of the results in Table 3 fall within the acceptable pH range (6.9–8.5). The highest power density and most effective treatment are found in substances with a high pH. These results agree with the prior studies (Behera and Ghangrekar 2017). At a pH of 7, the carbohydrate-to-nitrogen ratio is ideal for MFC (Chang and Herrmann 2018). Bacteria, however, have been found to thrive in environments with a pH between 6.3 and 7.8 (Zhu et al. 2010). Marashi routinely diluted wastewater by a factor of 10 to test the single-chamber MFC at pH values of 8.5, 7.0, and 5.4. These pH values were selected because they fall within the optimal range for methane-producing bacteria, and the highest power density was measured at 12.5 mW/m^2^ at a pH of 8.5, 7.5 mW/m^2^ at a pH of 7.0, and 4.3 mW/m^2^ at a pH of 5.4. The produced power was 40% higher at pH 8.5 than at pH 7.0 and 66% higher at pH 5.4. Yuan et al. (2016) state power density increases as pH rises. The power output trend at different pH levels indicates that an alkaline environment is preferable for developing electrogenic bacteria. Previous studies have shown that bacteria’s electrochemical interaction increases dramatically in alkaline conditions (Behera et al. 2011). The maximum power density was seen in alkaline circumstances (pH 8.5) due to the inactivation of acidogenic and methanogenic bacteria in favour of increased activity for electrogenic bacteria (Anthony Janicek and H. L. 2014; Ishaq et al. 2023).

**Table 3:** Reported studies on the performance of different types of MfCs with the use of landfill leachate as a substrate

S/N	Influent (mgNL^−1^)	Reactor type		Operating mode	Substrate type	Temperature (°C)	pH	HRT	Removal efficiency	Power density (PD)	Conclusion	Ref.
		Chamber	Install shape									
1	COD:12,900 BOD5:6300	Double chamber	Tubular	Continuous	Leachate effluent	30	7.74	4 days	COD:79% BOD5: 82%	PD: 1 mW/m^3^	The power output increased by 118 0% when the electrode surface area was expanded from 360 to 1080 cm^2^.	(Gálvez et al. 2009)
2	COD: 1022	Double chamber	Box	Batch	Leachate effluent	32	7.7–8.2	45 days	COD:78% colour: 77%	PD: 4.2 W/m^3^	The pyrrhotite-cathode MFC treated old-aged landfill leachate by a bioelectrochemically driven Fenton's reaction.	(Zhu et al. 2010)
3	COD:3480 TN:6033 N-NH3: 900	Single chamber	Tubular	Continuous	Leachate effluent	23 ± 2	7.4	46 days	COD:37% TN: 43%	PD:344 mW m^−3^ CE: 2%	The high salt content aided the MFC's performance, which increased power generation while lowering internal resistance. However, Ammonium was removed from the leachate due to ammonia loss or ammonium transfer via the membrane.	(Puig et al. 2011)
4	COD:5000	Double chamber	Tubular	Continuous	Leachate effluent	27	7.8	12 h	COD:45%	PD:1 mWm^−3^ CE: 57%		(Özkaya et al. 2013)
5	COD:3200 NH4+-N: 1150	Single chamber	Tubular	Batch	Leachate effluent	23	7.8±0.3	52 days	COD:16% ${\textrm{NH}}_4^{+}$-N:25% BOD: 74% TOC: 27%	PD: 824 mWm^−3^	Based on the leachate’s microbial characteristics, supportive and inhibiting bacteria may be present in landfill leachate for MFC operation.	(Damiano et al. 2014)
6	COD:12,300 TAN: 2900	Single chamber	Tubular	Batch	Leachate effluent	23	8.26	75 h	COD: 85%	PD: 1799 mWm^−3^ CE: 6.7%	The performance of the MFC with catalyst Pt exhibited high performance.	(Vázquez-Larios et al. 2015)
7	COD:17,500–22,600 NH4+-N: 1170–1490	Single Chamber	Tubular	Continuous	Leachate effluent	56	48 h	7.5 h	COD:89.1.6% ${\textrm{NH}}_4^{+}$-N:99.2%	PD: 2.71±0.09 Wm^−3^	COD removal was developed with A/O zones to achieve sequential nitrification–denitrification for ammonium removal. It was demonstrated that there is a high power density.	(Zhang et al. 2015)
8	Landfill leachate: 20L	Dual chamber	Cylindrical box	Fed-batch	Leachate effluent	35	6.5	30 days	Activated carbon: 96.6%Zinc electrode:66.6% Black carbon: 92.8%	PD_AC_: 0.75 mWm^−3^ PD_ZC_: 3.60 mWm^−3^ PD_BC_: 0.40 mWm^−3^	Activated carbon has the highest removal rate of ammonia, about 96.6%. However, zinc is a more appropriate choice for power generation.	(Alabiad et al. 2017)
9	COD:6842.1 NH_3_–N: 3521	Double chamber	Bottle box	Fed-batch	Leachate effluent	30	8.84	45 days	Chemical-Cathode MFC (CMFC) and Aerobic Bio-cathode MFC (ABMFC). COD and NH_3_ CMFC: 56.5%, 53.8% (ABMFC): 64.3%, 58.1%, respectively.	PD_CMFC_:699.0 mV:197.7mWm^−3^ PD_ABMFC_:459 mV:147.6 mWm^−3^	ABMFC exhibit high power density as well as effective treatment efficiency.	(Hu et al. 2017)
10	COD:3480	Single Chamber	Bottle	Batch	Leachate effluent	32	7.2	3.2 days	COD: 98.7%	PD:6.64 mW/m^3^	MFC exhibits high COD removal with maximum power density potential.	(Kumar et al. 2019)
11	NH_4_^+^N:1633 COD:5000	Supercapacitor single chamber	Cylindrical box	Fed-batch	Leachate effluent	27	8.2	18 days	COD: 59.4%; NH_4_-N :78.2%	CD:2.1×10^−4^ A cm^−2^ at 60% LC: PD:298 ± 22 mWm^-2^	Spontaneous landfill leachate treatment and bioelectricity generation were demonstrated.	(Cai et al. 2020)
12	NH_4_-N: 2514 COD: 20,055	Single chamber	Cylindrical box	Fed-batch	Leachate effluent	25	7.8	3 days	COD: 86% NH_4_-N: 89.4%	Voltage: 0.39 V	Algal assimilation with higher concentrations of NH3 and COD	(Elmaadawy et al. 2020a)
13	COD:1503 N-NH_4_^+^:526	Three-chamber	Cylindrical box	Fed-batch	Leachate effluent	25.0± 2.9	6.90 ± 0.60	30 days	COD:79±2% N-NH_4_:72±6% DOC: 43 ± 3%	PD:34 mW/m^2^. Voltage: 463 ± 41 mV	The electrochemical membrane bioreactor compartments showed distinct microbial communities and inferred metabolic pathways	(Pierangeli et al. 2021)
14	COD:500 NH4-N:1000	0smotic MFCs chamber	Bottle box	Batch	Leachate effluent	36± 3 °C.	8.3± 0.0	3 days	TN:74% NH_4_-N:70%	PD: 0.44 Wm^−2^	OsMFC showed the highest electricity production and most efficient pollutant removal under both operation modes	(Jiang et al. 2021)
15	COD:1022	Double chamber	Rectangular	Batch	Leachate effluent	Room temperature	-	9.4 h	COD:78±1.2%	MPD: 4.2 W/m^3^		(Li et al. 2014)

## Some applications of MFC in landfill leachate treatment

To determine how well a design works, it must be moved from the lab scale to the pilot size in a planned way. Before putting in place large-scale applications, this method will help find any problems that might come up. MFC has been used to treat many substrates, from simple synthetic wastewater to difficult real wastewater (Pandey et al. 2016). Table 2 displays the instances of MFC using actual landfill leachate gathered from the literature. Inputs, reactor type (chamber installation shape), mode of operation, substrate, temperature, pH, HRT, removal capacity, energy production, and outcome were the key classifications. MFCs can be made in a variety of shapes, including a tube, a box, or a flat design. In almost all cases, MFCs were operated in a state of constant flow.

In many cases, much power can be generated in batch mode. Since the batch mode requires the replacement of the substrate to provide constant power output, the continuous-flow method is more practical than the batch mode when it comes to electricity production (X. Wang et al. 2008). The inputs of MFCs are flexible, and there is typically a linear relationship between energy production and COD concentration (Juang et al. 2011). Therefore, the potential power yield is affected by both the type of substrate and the concentration. Since bacteria can efficiently utilise a finite amount of organic matter or wastewater (Yu et al. 2021), the MFC's energy efficiency may significantly affect the wastewater parameters when using real wastewater. Due to its high COD concentration, early studies focused primarily on non-domestic wastewater, such as landfill leachate. Power density, TAN, COD, and biodegradability all have a direct linear relationship. The performance of MFCs could be improved using wastewater with a high organic matter content. The power density of MFCs has grown substantially in recent years (Pham et al. 2009). By adjusting for anodic volume, the maximum power density of a single MFC increased to 200 W/m^3^, while that of stacked MFCs increased to 250 W/m^3^ (Deeke et al. 2015). While the average power density was higher when MFCs were fed synthetic wastewater, it dropped when fed actual landfill effluent due to limiting factors like the lack of readily available electron acceptors other than the anode (F. Lu et al. 2009). Most MFCs treating real wastewater have a power density of less than 20 W/m^3^ (Table 2), regardless of chamber size or form, installation method, mode of operation, or wastewater type. Power density rises with increasing electrode area per unit volume in a reactor (Cheng et al. 2011). This may improve electron transport from the anode to the cathode and stimulate bacterial growth. Power densities of up to 4240 mW/m^3^ were achieved by the rectangular box-type MFC, which is significantly lower than the previously claimed figure of 20 W/m^3^ (Ahn and Logan 2010). However, the flat-type MFC with a low volumetric ratio has the highest Potential Density because of the short inter-electrode distance and the broad contact surface between the anode and cathode (Kim et al. 2019). Although, in theory, 1 kg of COD could be transformed into 3.86 kWh of energy (Richter et al. 2008), this is unlikely to ever happen in practice. MFCs are still seen as a practical way to extract useful energy from wastewater, as was previously mentioned. However, due to MFCs’ lower energy generation and energy recovery, their practical potential as an energy source remains insufficient. Research is needed into the true inhibitory concentration and internal resistance that may decrease energy dissipation, as well as the material (electrode and catalyst) and reactor layout (increased AV ratio, short inter-electrode distance, and high contact area).

## Problems and future prospects

The management of landfill leachate poses numerous difficulties due to its exceptional qualities, including elevated conductivity and the existence of environmentally hazardous elements. In this regard, MFC technology has emerged as a promising solution to effectively and stably treat this leachate. Nevertheless, it is vital to tackle certain ongoing challenges that demand considerable attention in the near future. One of the primary limitations of MFC technology is the high power supply voltage and energy consumption, as reported by Fernandes et al. in 2015. Luckily, different power sources like solar, wind, and biomass energy, as pointed out by Nie et al. (2020), Yuan et al. (2022), and Jeon et al. (2016), generated higher power output than MFCs. The integration of MFCs with alternative energy sources such as solar, wind, and biomass energy holds the potential to curtail energy expenditures. Despite the positive performance of MFC technology in landfill leachate treatment, issues like high effluent conductivity persist, which calls for pre-discharge desalination.

Additionally, further research is needed to investigate the endpoint of toxicity reduction during MFC electrolysis, as this can help save on treatment costs based on pollutant properties. Therefore, it is imperative to delve deeper into the transformation and toxicity alterations of organic matter during the MFC process. Furthermore, the potential use of the resulting harmless organic matter for resource applications, such as irrigation or soil improvement, holds significance for local carbon cycle reconstruction and organic carbon resource conservation. Considering the relationship between treatment cost and efficacy, which encompasses pollution load reduction and toxicity reduction in landfill leachate, the integration of MFC and Advanced conventional treatment methods has been proposed. These procedures, like electroFenton, photoelectro-Fenton, electro-catalytic ozonation, and electrochemical-persulfate oxidation, are designed to increase the rate of mineralization of landfill leachate while minimizing unnecessary reaction time. However, these methods face challenges in *NH*_3_ − *N* removal and are pH dependent. Therefore, they can be effectively integrated as part of electrochemical degradation processes, either for pre-treatment or advanced treatment. Additionally, the construction of anaerobic MFC systems shows the potential to reduce pollution loads and recover energy. However, additional assessment is necessary to ascertain the optimal operational parameters and synergistic combinations with other technologies. In light of the strengths and limitations of various electrochemical processes and integration approaches, the amalgamation of different techniques becomes a constructive strategy for developing a high-efficiency treatment process for landfill leachate. The prospects for landfill leachate treatment show promise. Ongoing research endeavours aim to enhance the performance of MFCs, incorporating modifications in operational parameters, reactor designs, and electrode materials. Additionally, the exploration of resource recovery and the circular economy, as well as advancements in electrochemical technology, afford avenues for more efficient and cost-effective treatment methodologies. The amalgamation of diverse electrochemical procedures and technologies, in conjunction with the integration of biological systems, can further augment treatment efficacy.

## Conclusion

This overview sheds light on how far we’ve come in understanding the Microbial Fuel Cell (MFC) technology, its key components, the significance of MFC design and configuration, and their effect on overall performance, as well as the problems and constraints associated with MFC scaling. The significant removal efficiency was shown for COD, BOD, colour, TN, and ${\textrm{NH}}_4^{+}$-N. COD removal efficiency ranged from 16 to 98.7% across investigations, and those for ${\textrm{NH}}_4^{+}$-N went from 25 to 99.2%. Environmental variables affected MFC performance, including pH, substrate type, salt concentration, and temperature. Longer lengths of operation were frequently correlated with greater removal efficiencies. Depending on the MFC’s setup and operating circumstances, the power density (how much energy is produced per unit volume) can vary from 0.44 to 799 mW/m^3^. One study indicated that a doubling of electrode surface area resulted in a 118% increase in power production.

In addition, the investigations highlighted the effect of varying chamber types (single and double) and installation designs (tubular, box, cylindrical box) on MFC performance. Electrode materials, including pyrrhotite, activated carbon, and zinc, all impacted how much energy could be produced and how much pollution could be cleaned up. MFC is useful in numerous trials for treating leachate, among other things. This literature review examines a synopsis of the many MFC treatment strategies and technologies developed for landfill leachate. Although the literature on MFCs’ use in landfill leachate treatment shows that the technology is effective at removing organic and ammonia compounds, its performance is hindered by several factors, including substrate concentration, refractory inhibition, high installation costs, electrode type, design and configuration, membrane material, and operating conditions. However, as shown in (Table 3), the age type of landfill leachate in response to operational conditions that could improve the transition from laboratory to industrial scale has not been examined. Upscaling due to low power out is a major issue in the current realm of MFC research. Nevertheless, Table 3 shows that it is still a promising pollutant removal and power generation technology. This literature study also found that there have been surprisingly few investigations on the feasibility of using landfill leachate as a substrate for efficient power generation and treatment. This may be due to the effluent’s complicated composition. To determine the best method for using it, more research is required. To scale up and increase the system's performance, the authors of this review study suggest using engineering application tools to optimize the operating parameters. The effect on ${\textrm{NH}}_4^{+}$-N efficiency in response to operating conditions of various technique combinations should be carefully evaluated.

## Data Availability

The manuscripts’ data is contained in the text.
